# Five New *Phyllachora* Species from Tar Spot Fungi on Poaceae in Sichuan China

**DOI:** 10.3390/jof11030208

**Published:** 2025-03-07

**Authors:** Pengwei Su, Yuechi Liao, Yan Jin, Yanpeng Chen, Asanka Madhushan, Jian-Kui Liu, Sajeewa S. N. Maharachchikumbura

**Affiliations:** School of Life Science and Technology, Center for Informational Biology, University of Electronic Science and Technology of China, Chengdu 611731, China; pengweisu@163.com (P.S.); yuechiliao@std.uestc.edu.cn (Y.L.); jinyan4759@163.com (Y.J.); yanpengch@outlook.com (Y.C.); asankakwm@gmail.com (A.M.)

**Keywords:** five new species, biotrophs, fungal taxonomy, multi-locus phylogeny, Poaceae pathogens, *Sordariomycetes*

## Abstract

Tar spot is a prevalent fungal disease characterized by shiny black spots on the leaves, stems, and fruits of various plants. It is typically caused by members of the family Phyllachoraceae, which consists of biotrophic and obligate plant parasitic fungi. During field investigations of tar spot fungi in Sichuan Province, China, 70 fungal samples associated with tar spots belonging to the family Poaceae were collected from 13 different locations. Through morphological studies and multi-locus phylogenetic analysis of combined ITS, SSU, and LSU datasets, the collected samples were classified into eight *Phyllachora* species. Among these, five were identified as new species (*Phyllachora cylindricae*, *P. festucae*, *P. luzhouensis*, *P. palmifoliae*, and *P. wenchuanensis*), and two represented new host records (*P. chongzhouensis*, *P. panicicola*). The new species are accompanied by descriptions and illustrations, while their characteristics are discussed in relation to comparable taxa. Additionally, *P. yuanjiangensis* is synonymized under *P. xinpingensis*. These findings enhance our understanding of tar spot fungi in Sichuan and, given that *Phyllachora* species are important pathogens of plants in the Poaceae family, establish a foundation for further research to better understand their potential impacts on agriculture and the local ecology.

## 1. Introduction

The genus *Phyllachora* (Phyllachoraceae, Phyllachorales) comprises biotrophic, obligate fungi that are widely distributed in tropical and subtropical regions, primarily parasitising the leaves and stems of Poaceae plants [1,2,3]. As a major plant pathogen, most *Phyllachora* species infect plant hosts and produce raised, melanized structures known as stromata, commonly referred to as tar spots [4]. These species are important plant pathogens, leading to diseases that severely affect the health and productivity of crops, ornamental plants, and forage grasses [5,6]. For instance, *Phyllachora maydis*, the pathogen responsible for tar spot disease in maize, was first identified in North America in 2015 [7]. Since its discovery, it has spread across the United States and Canada, resulting in reduced grain yield and diminished quality of silage, stover, and husks. In 2021, the economic losses in the United States attributed to tar spot were estimated at 5.97 million metric tons (235 million bushels) in yield reductions, equating to approximately 1.2 billion U.S. dollars (Crop Protection Network Disease Loss Calculator; https://loss.cropprotectionnetwork.org/, accessed on 5 December 2024) [8].

The family Phyllachoraceae was introduced by Theissen and Sydow [9] in 1915 and currently includes 54 genera, with *Phyllachora* as the type genus [10,11,12]. *Phyllachora* is the largest genus within the family Phyllachoraceae and has presently 1520 epithets in Index Fungorum (http://www.indexfungorum.org/ (accessed on 18 January 2025)). After excluding species that have been synonymized or transferred to other genera, approximately 990 species remain, although many still exhibit cases of synonymy. Species of this genus are morphologically characterized by conspicuous black clypeate and stromata on the surface or within the leaf tissue, typically appearing oblong to elongated, fusiform, or irregular, surrounded by yellow or pale brown necrotic lesions [13,14]. The ascomata are typically globose, oval, or irregular and are situated subcuticular or immersed within the host tissue, accompanied by numerous branched paraphyses that are slightly longer than the asci [15,16]. The asci are cylindrical, hyaline, short to medium pedicellate, containing four or eight ascospores [13,17]. The ascospores are arranged in monostichous, diplostichous, or irregular patterns [14]. They are round, ovoid, elliptical, or fusiform, hyaline or pale yellow, with smooth or verrucose and may sometimes possess a gelatinous sheath [13,16].

Sichuan Province is recognized as a biodiversity hotspot, showcasing an impressive variety of fungal species [18,19]. Numerous studies have documented the region’s abundance of tar spots, particularly impacting Poaceae crops, with both newly identified and previously known species of *Phyllachora* being recorded [14,16,20]. Additionally, Sichuan Province, especially the Yangtze River Delta, is a crucial area in China for cultivating Poaceae crops, including significant quantities of rice, maize, wheat, barley, foxtail millet, and broomcorn millet [21,22]. Also, numerous reports indicate the persistent threat of pathogenic fungi to crop production globally. These pathogens have shown a remarkable ability to evolve genetically, increasing their virulence under diverse selection pressures, often transitioning from natural environments to agricultural contexts [23]. Therefore, given the significant prevalence of tar spots, Sichuan presents a considerable challenge for local agriculture. This situation highlights the significance of investigating the diversity and effects of *Phyllachora* species in Poaceae plants within natural environments in Sichuan province. Therefore, in this study, we present findings from an ongoing survey of tar spot fungi in Sichuan Province, China, conducted from October 2021 to September 2023. A total of 70 specimens were collected from 13 sampling sites across a diverse range of Poaceae plants. Combining morphological studies with multi-gene phylogenetic analysis, we identified eight species, comprising five new species and two new host records. Detailed morphological descriptions, illustrations, and sequence data for these species are provided, offering valuable insights into the diversity within the genus *Phyllachora*.

## 2. Materials and Methods

### 2.1. Sample Collection, Morphological Examination, and Preservation

A survey of tar spot fungi was conducted in Sichuan Province, China, from October 2021 to September 2023. Diseased graminaceous plants were collected from 13 sampling sites across seven regions, including Chengdu City, Meishan City, Luzhou City, Mianyang City, Guangyuan City, Aba Tibetan and Qiang Autonomous Prefecture, and Ganzi Tibetan Autonomous Prefecture. More detailed information is provided in Supporting Information Supplementary Table S1. These fresh specimens were carefully stored in paper envelopes and subsequently transported to the laboratory for further examination. All specimens were preserved in a refrigerator at 4 °C for extended periods. The macromorphological observations and recordings were conducted using a Nikon SMZ800N stereomicroscope with a Nikon DS-Fi3 camera (Nikon Corporation, Tokyo, Japan). Hand sections were prepared under the stereomicroscope and mounted on slides with sterile water. The fungal microstructures were then photographed using the Nikon DS-Ri2 microscope camera (Nikon Corporation, Tokyo, Japan) attached to a Nikon Eclipse Ni-U microscope (Nikon Corporation, Tokyo, Japan). All measurements were recorded using Nikon NIS-Elements D 5.21 (Nikon Corporation, Tokyo, Japan) software, and the images were processed with Adobe Photoshop version 22.0 (Adobe Inc., San Jose, CA, USA). The holotypes were deposited in the Herbarium of Cryptogams at the Kunming Institute of Botany, Academia Sinica (HKAS) in Kunming, China, or the Herbarium of the University of Electronic Science and Technology (HUEST) in Chengdu, China. The taxonomic descriptions of the new taxa are deposited in MycoBank.

### 2.2. DNA Extraction, PCR Amplification, and Sequencing

Fungal genomic DNA was extracted from ascomata using the *SteadyPure* Plant Genomic DNA Extraction Kit (AG21026, Accurate Biotechnology, Changsha, Hunan, China) according to the manufacturer’s protocol. The DNA was stored at −20 °C for long-term storage. The three barcodes, which include the nuclear ribosomal internal transcribed spacer (ITS: ITS1-5.8S-ITS2), the partial nuclear ribosomal small subunit rRNA (SSU) and the partial nuclear ribosomal large subunit rRNA (LSU) were amplified by polymerase chain reaction (PCR). The pairwise primers were ITS9mun and ITS4_KYO1 [24] for ITS, LR0R [25] and LR5 [26] for LSU, and PNS1 [27] and NS41 [28] for SSU. The final PCR system was 30 µL, containing 15 μL PCR Master Mix (CoWin Biosciences, Taizhou, China), 11 µL of double-distilled water (ddH_2_O), 1 μL each forward and reverse primers (10 μM), and 2 μL DNA template. The PCR products were analyzed by electrophoresis in 1% agarose gels. Sanger sequencing was conducted by Sangon Biotech (Shanghai, China).

### 2.3. Phylogenetic Analyses

The consensus sequences were generated by manual editing, trimming, and assembling raw Sanger sequencing chromatograms using SeqMan Pro version 11.1.0 (DNASTAR, Inc., Madison, WI, USA). Barcode sequences of the family Phyllachoraceae species currently available in GenBank, along with the outgroup taxon *Telimena bicincta* (MM 133), were downloaded from the NCBI nucleotide database using the R package Analysis of Phylogenetics and Evolution (APE) version 5.8-1 [29]. Multiple sequence alignments were performed using MAFFT version 7.310 [30] with options “--maxiterate 1000 --genafpair --adjustdirectionaccurately” and the alignment results were further trimmed using trimAl version 1.4 [31] with the option “-gapthreshold 0.5”, which only allows 50% of taxa with a gap in each site. The best-fit nucleotide substitution models for each alignment dataset were selected using ModelFinder based on the Corrected Akaike Information Criterion (AICc) [32].

DNA barcode sequence datasets from the trimmed alignments were concatenated using an in-house Python (version 3.11.10) script. Maximum Likelihood (ML) and Bayesian Inference (BI) analyses were performed using the combined datasets. The ML phylogenetic tree was generated using IQ-TREE version 2.2.2.6 [33], with its topology evaluated through 1000 ultrafast bootstrap replicates. BI analysis was conducted using parallel MrBayes version 3.2.7a [34]. Two independent analyses were conducted with 20 million generations and four chains. The initial 25% of sampled trees were discarded as burn-in. Convergence of the Markov Chain Monte Carlo (MCMC) runs was confirmed using Tracer version 1.7.1 [35], ensuring all adequate sample size (ESS) values exceeded 200. The ML tree was annotated using TreeAnnotator version 2.7.3, which was implemented in BEAST version 2.7.3 [36] based on MrBayes MCMC trees without discarding burn-in and applying no posterior probability limit. Finally, the ML tree was visualized with ggtree [37] and further refined using Adobe Illustrator version 22.0.0 (Adobe Inc., CA, USA).

## 3. Results

### 3.1. Molecular Phylogeny

**Figure 1:**
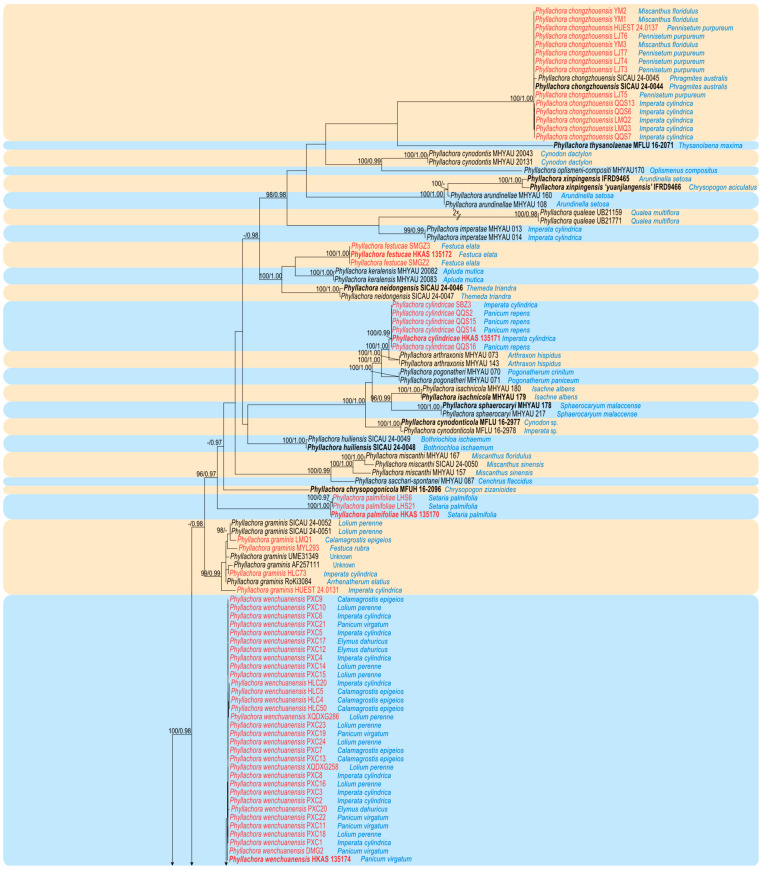

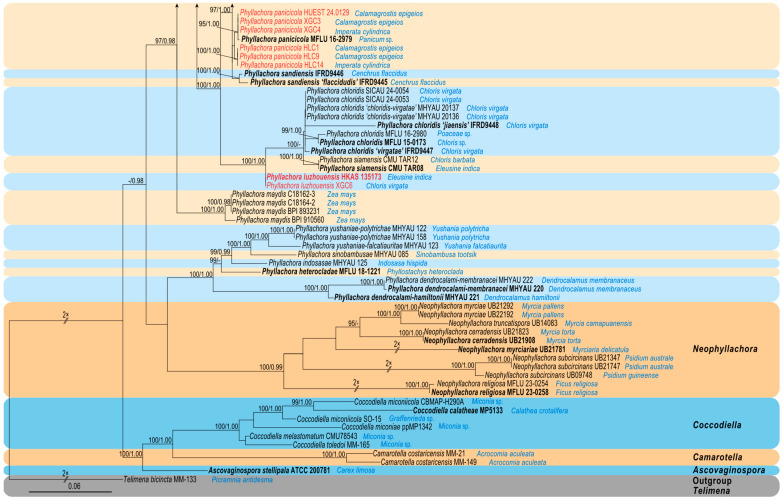
A phylogram of the best-scoring ML consensus tree based on the concatenated dataset (LSU-SSU-ITS). The type specimens are indicated in bold, while the specimens collected in this study and synonymized taxa are highlighted in red, accompanied by the host species corresponding to their specimen number. The ML ultrafast bootstrap values/Bayesian PP greater than 95%/0.95 are displayed at the respective nodes. The tree is rooted with *Telimena bicincta* MM-113.

The newly generated sequences were deposited in GenBank, and the accession numbers are listed in Table 1. The concatenated dataset consists of three loci, namely LSU, SSU, and ITS, obtained from 157 specimens (including 70 specimens collected in this study) belonging to the family Phyllachoraceae, with *Telimena bicincta* (MM 133) as the outgroup. The dataset comprises a total of 2369 characters (LSU: 1–819; SSU: 820–1825; ITS: 1826–2369), including gaps, and consists of 1542 distinct patterns, 995 parsimony-informative sites, 424 singleton sites, and 950 constant sites. The best-fit evolution models for ML and MrBayes phylogenetic analysis were GTR+F+I+G4 for the LSU, K2P+G4 for the SSU, and SYM+I+G4 for the ITS. The best-scoring ML tree (lnL = –28,490.650), with ultrafast bootstrap values from ML analyses and posterior probabilities from the MrBayes analysis, is depicted at the node in Figure 1. Phylogenetic analyses demonstrated that our newly collected 70 specimens clustered into eight distinct clades within the genus *Phyllachora*. These comprise five new species (*Phyllachora cylindricae*, *P. festucae*, *P. luzhouensis*, *P. palmifoliae*, and *P. wenchuanensis*) and three known species (*P. chongzhouensis*, *P. graminis*, and *P panicicola*).

### 3.2. Taxonomy

#### 3.2.1. *Phyllachora chongzhouensis* Q.R. Sun, X.L. Xu & C.L. Yang. J. Fungi 10 (No. 588): 8 (2024). Figure 2

*MycoBank*: MB 902115

*Parasitic* on leaves and stems of Poaceae. Tar spots 2–4 mm wide, oval, oblong or elongated, black, shiny, carbonaceous. **Sexual morph**: *Ascomata* 295–370 μm high, 250–300 μm diam ($\bar{x}$ = 320 × 280 μm, n = 15), permeating the leaf tissue, covering its entire thickness, like black nevus, domed above the leaf surface, subglobose, ellipsoidal, ostiole inconspicuous, scattered, solitary to gregarious, black, unicellular or multilocular. *Peridium* 40–120 μm wide ($\bar{x}$ = 83 μm, n = 20), composed of dark brown to black cells of textura angularis with slender, darker cells of outer layer, and large, slightly paler cells of interlayer. *Paraphyses* 1.5–5 μm wide ($\bar{x}$ = 3 μm, n = 20), hyaline, filament-like, and without any branches, septate, not constricted at septate, possessing lengths that surpass the asci, with apically narrowing ends, originating from the innermost basal and lateral walls. *Asci* 92–163 × 17–31 μm ($\bar{x}$ = 125 × 23 μm, n = 35), 8-spored, cylindrical, straight or curved, short to medium pedicellate, apex obtuse to rounded, hyaline. *Ascospores* 17–25 × 10–15 μm ($\bar{x}$ = 20 × 12 μm, n = 40), uniseriate, oblique, sometimes irregularly arranged, subglobose to ellipsoidal, rounded at the ends, aseptate, verrucous, hyaline, with a gelatinous sheath. **Asexual morph**: Undetermined.

**Figure 2:**
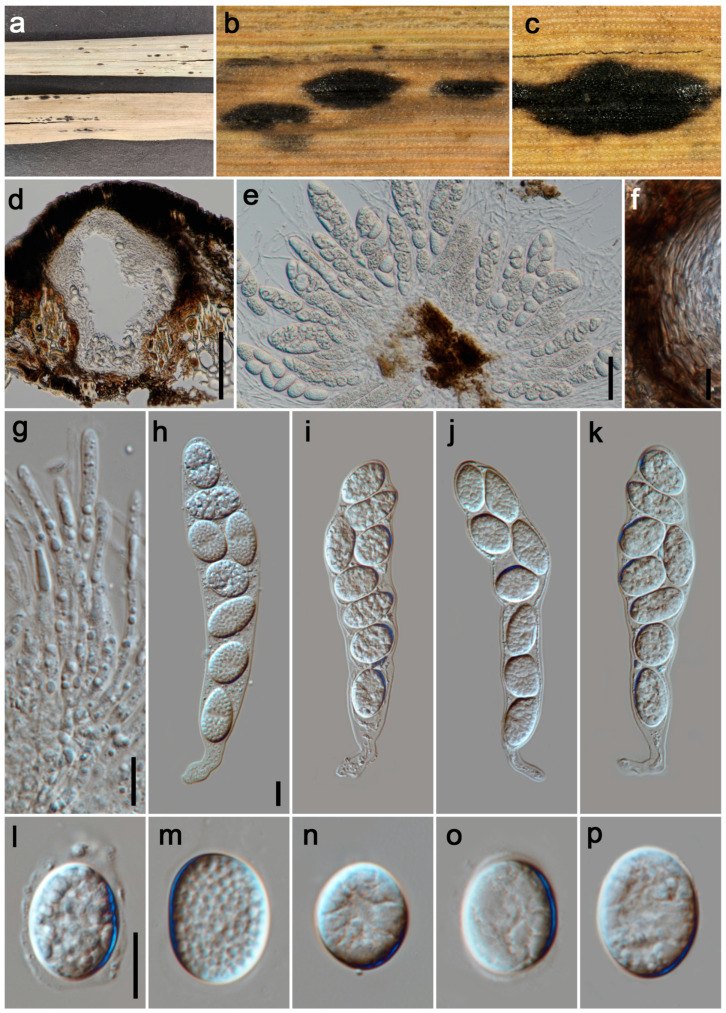
***Phyllachora chongzhouensis*** (HUEST 24.0137). (**a**) Black spots on *Pennisetum purpureum* (Poaceae); (**b**,**c**) Stromata; (**d**) Vertical section of ascomata; (**e**) Asci and paraphyses; (**f**) Peridium; (**g**) Paraphyses; (**h**–**k**) Asci; (**l**–**p**) Ascospores. Scale bars: 100 μm (**d**), 20 μm (**e**), 10 μm (**f**–**h**,**l**). Scale bar of (**h**) applies to (**i**–**k**); Scale bar of (**l**) applies to (**m**–**p**).

*Host distribution: Imperata cylindrica*, *Miscanthus floridulus*, *Pennisetum purpureum*, *Phragmites australis* [16].

*Material examined*: China, Sichuan Province: Chengdu City, Wenjiang District, Lujiatan Wetland (30°41′38″ N, 103°46′5″ E, elevation 495 m), on stems and leaves of *Pennisetum purpureum* (Poaceae), 3 September 2023, P.W. Su, LJT2 (HUEST 24.0137); ibid., LJT3, LJT4, LJT5, LJT6, LJT7. Guangyuan City, Jiange County, Lanmaqiang (32°0′42″ N, 105°24′37″ E, elevation 655 m), on stems and leaves of *Imperata cylindrica* (Poaceae), 24 May 2023, P.W. Su, LMQ2; ibid., LMQ3. Mianyang City, Zitong County, Qiqu Mountain (31°41′37″ N, 105°11′8″ E, elevation 725 m), on stems and leaves of *Imperata cylindrica* (Poaceae), 25 May 2023, P.W. Su, QQS6; ibid., QQS7, QQS13. Chengdu City, Chongzhou City, Yangma Wetland Park (30°39′18″ N, 103°45′3″ E, elevation 492 m), on stems and leaves of *Miscanthus floridulus* (Poaceae), 27 May 2023, P.W. Su, YM1; ibid., YM2, YM3.

*Notes*: In the phylogenetic tree (Figure 1), our 14 specimens (HUEST 24.0137, LJT3–LJT7, LMQ2, LMQ3, QQS6, QQS7, QQS13, YM1–YM3) clustered together with the known species *P. chongzhouensi* (SICAU 24-0044 and SICAU 24-0045). *Phyllachora chongzhouensis* was first identified parasitizing the leaves of *Phragmites australis* in China [16]. Morphologically (Table 2), there are no significant differences between our specimens and the type species of *P. chongzhouensis*. Based on morphological diagnosis and phylogenetic analyses, our 14 collections are identified as *P. chongzhouensis*, marking the first records of this species from *Imperata cylindrica*, *Miscanthus floridulus*, and *Pennisetum purpureum*.

#### 3.2.2. *Phyllachora cylindricae* P.W. Su & Maharachch., sp. Nov. Figure 3

*MycoBank*: MB 852909

*Etymology*: Name reflects the epithet of the host plant, *Imperata cylindrica*, from which the fungus was collected.

*Parasitic* on leaves and stems of Poaceae. Tar spots 1–2 mm wide, oblong, fusiform, black, carbonaceous. **Sexual morph**: *Ascomata* 200–270 μm high, 260–295 μm diam ($\bar{x}$ = 235 × 280 μm, n = 15), distributed throughout the leaf tissue, spanning its full thickness, domed above the leaf surface, subglobose or ellipsoidal, like black nevus, ostiole inconspicuous, scattered, solitary to gregarious, black, shiny, unicellular or multilocular. *Peridium* 55–70 μm wide ($\bar{x}$ = 61 μm, n = 15), an outer region composed of brown to black cells of *textura angularis*, inner layers consisting of multiple hyaline, thin-walled strata, flattened fungal cells. *Paraphyses* 1.5–3.5 μm wide ($\bar{x}$ = 2.5 μm, n = 20), hyaline, abundant, thread-like and unbranched, aseptate, longer than the asci, with tapering apices, originating from the inner basal and lateral walls. *Asci* 80–128 × 10–16 μm ($\bar{x}$ = 95 × 13 μm, n = 35), 8-spored, cylindrical, straight or curved, short to medium pedicellate, apex obtuse to rounded, hyaline. *Ascospores* 11–15 × 6–8 μm ($\bar{x}$ = 13 × 7 μm, n = 40), uniseriate, sometimes oblique, tear-shaped, ovoid to ellipsoidal, rounded at the base, tapered at the apex, aseptate, smooth, hyaline, with a gelatinous sheath. **Asexual morph**: Undetermined.

*Host distributions*: *Imperata cylindrica*, *Panicum repens*.

*Material examined*: China, Sichuan Province, Mianyang City, Zitong County, Shuangban Town, 31°49′40″ N, 105°3′11″ E, elevation 655 m, on stems and leaves of *Imperata cylindrica* (Poaceae), 25 May 2023, P.W. Su, SBZ2 (HKAS 135171, holotype; HUEST 24.0130, isotype); ibid., SBZ3. Mianyang City, Zitong County, Qiqu Mountain, 31°41′37″ N, 105°11′8″ E, elevation 725 m, on stems and leaves of *Panicum repens* (Poaceae), 25 May 2023, P.W. Su, QQS2, QQS14; ibid., QQS15, QQS16.

**Figure 3:**
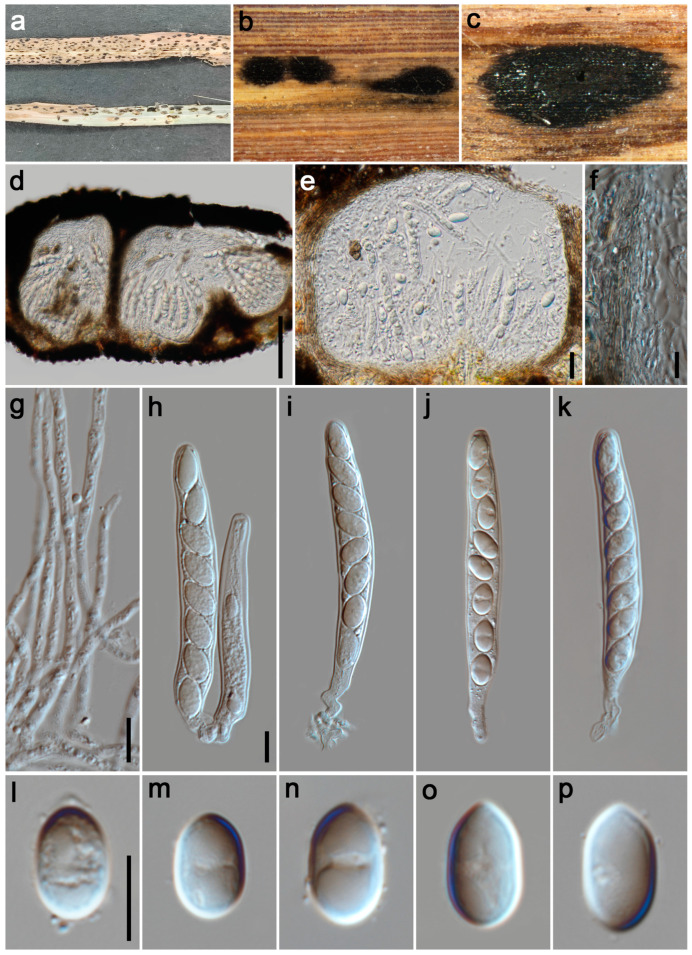
***Phyllachora cylindricae*** (HKAS 135171, holotype). (**a**) Black spots on *Imperata cylindrica* (Poaceae); (**b**,**c**) Stromata; (**d**,**e**) Vertical section of ascomata; (**f**) Peridium; (**g**) Paraphyses; (**h**–**k**) Asci; (**l**–**p**) Ascospores. Scale bars: 100 μm (**d**), 20 μm (**e**), 10 μm (**f**–**h**,**l**). Scale bar of (**h**) applies to (**i**–**k**); Scale bar of (**l**) applies to (**m**–**p**).

*Notes:* The phylogenetic tree (Figure 1) revealed that specimens HKAS 135171, SBZ3, QQS2, QQS14, QQS15, and QQS16 form a clade that is sister to *Phyllachora arthraxonis* (MHYAU 073 and MHYAU 143), with high statistical support values (100% ML, 1.00 PP)*. Phyllachora arthraxonis* was first collected by Hennings et al. [38] in 1904 from the leaves of *Arthraxon ciliaris*, and its molecular data were subsequently deposited to the NCBI by Li et al. [15]. Morphologically, the asci (79.26–128.26 × 10.34–15.98 μm vs. 35–45 × 8–12 μm) and ascospores (10.67–15.27 × 6.45–8.27 μm vs. 8–11 × 4–5 μm) of HKAS 135171 are significantly larger than those of the type specimen of *P. arthraxonis* [38]. Additionally, the BLASTn analysis of HKAS 135171 and MHYAU 073 shows a 98% identity (525/534, 1 gap) using ITS. Therefore, based on morphological characteristics and phylogenetic analyses, we describe our collections as a new species of *Phyllachora.*

#### 3.2.3. *Phyllachora festucae* P.W. Su & Maharachch., sp. Nov. Figure 4

*MycoBank*: MB 852908

*Etymology*: Name reflects the host genus, *Festuca*, from which the fungus was collected.

*Parasitic* on leaves and stems of *Festuca elata*. Tar spots 2–4 mm wide, on the upper leaf surface, oval, oblong or elongated, black, shiny, sometimes yellow to brown stripe at the edge of tar spots. **Sexual morph**: *Ascomata* 205–365 μm high, 205–720 μm diam ($\bar{x}$ = 310 × 550 μm, n = 15), permeating the leaf tissue, encompassing its total thickness, domed above the leaf surface, subglobose or ellipsoidal, like black nevus, ostiole inconspicuous, scattered, solitary to gregarious, black, shiny, irregularly multilocular. *Peridium* 55–95 μm wide ($\bar{x}$ = 75 μm, n = 20), an outer region composed of brown to dark brown cells of *textura angularis*, the inner layers are formed by multiple hyaline, thin-walled layers, flattened fungal cells. *Paraphyses* 2–3 μm wide ($\bar{x}$ = 2.5 μm, n = 20), hyaline, filiform, unbranched, aseptate, exceeding the length of the asci, featuring tapering apices that emerge from the inner basal and lateral walls. *Asci* 135–222 × 12–18 μm ($\bar{x}$ = 163 × 15 μm, n = 35), 8-spored, long and cylindrical, short pedicellate, apex obtuse to rounded, hyaline. *Ascospores* 17–27 × 8–12 μm ($\bar{x}$ = 23 × 10 μm, n = 40), uniseriate, sometimes overlapping and oblique, long ellipsoidal to fusiform, acute at the ends, hyaline, with a gelatinous sheath. **Asexual morph**: Undetermined.

*Host distribution*: *Festuca elata*.

*Material examined*: China, Sichuan Province, Aba Tibetan and Qiang Autonomous Prefecture, Wenchuan County, Shuimoguzhen, 30°56′4″ N, 103°25′19″ E, elevation 902 m, on stems and leaves of *Festuca elata* (Poaceae), 28 May 2023, P.W. Su, SMGZ1 (HKAS 135172, holotype; HUEST 24.0132, isotype); ibid., SMGZ2, SMGZ3.

*Notes*: Multi-gene phylogenetic analysis revealed that specimens HKAS 135172, SMGZ2, and SMGZ3 clustered together in a distinct clade, forming a sister group to *Phyllachora keralensis* (MHYAU 20082, MHYAU 20083). The BLASTn analysis of HKAS 135172 and MHYAU 20082 shows only 83% identity (426/515, 19 gaps) using ITS. Additionally, our specimens HKAS 135172 have significantly larger asci (135.54–222.89 × 12.02–17.82 μm vs. 52–68 × 7–9.5 μm) and ascospores (17.02–27.17 × 8.59–12.46 μm vs. 10–14 × 5–6.5 μm) compared to the type specimen of *P. keralensis* [39]. Based on differences in morphological features, molecular sequences, and multi-gene phylogenetic analysis, we describe the newly collected specimens HKAS 135172, SMGZ2, and SMGZ3 as a new species, *Phyllachora festucae*.

**Figure 4:**
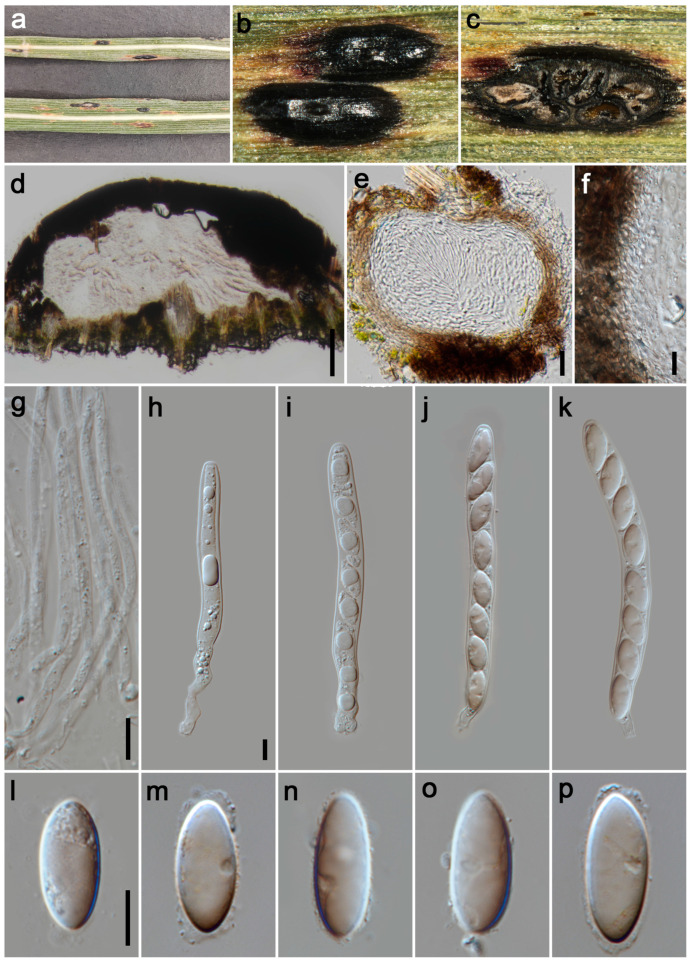
***Phyllachora festucae*** (HKAS 135171, holotype). (**a**) Black spots on *Festuca elata* (Poaceae); (**b**) Stromata; (**c**) Horizontal section of ascomata; (**d**,**e**) Vertical section of ascomata; (**f**) Peridium; (**g**) Paraphyses; (**h**–**k**) Asci; (**l**–**p**) Ascospores. Scale bars: 100 μm (**d**), 20 μm (**e**), 10 μm (**f**–**h**,**l**). Scale bar of (**h**) applies to (**i**–**k**); Scale bar of (**l**) applies to (**m**–**p**).

#### 3.2.4. *Phyllachora graminis* (Pers.) Fuckel, Jahrb. Nassauischen Vereins Naturk. 23–24: 216 (1870). Figure 5

*MycoBank*: MB 200927

**Figure 5:**
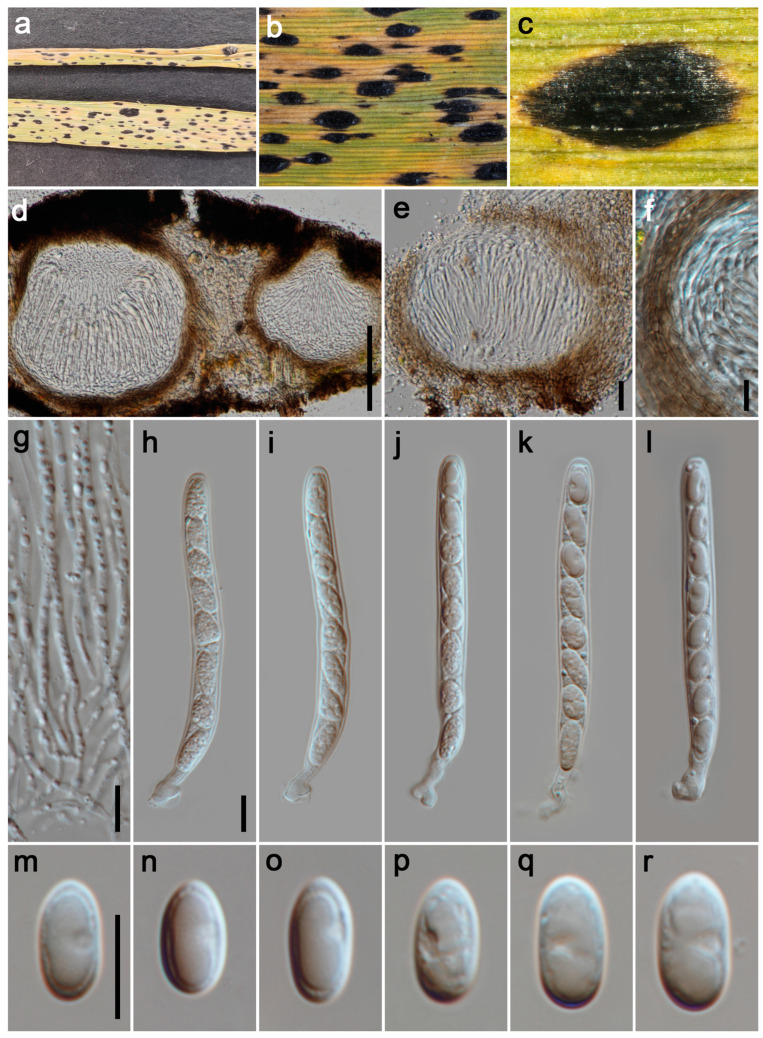
***Phyllachora graminis*** (HUEST 24.0131). (**a**) Black spots on *Imperata cylindrica* (Poaceae); (**b**,**c**) Stromata; (**d**,**e**) Vertical section of ascomata; (**f**) Peridium; (**g**) Paraphyses; (**h**–**l**) Asci; (**m**–**r**) Ascospores. The scale bars: 100 μm (**d**), 20 μm (**e**), 10 μm (**f**–**h**,**m**). Scale bar of (**h**) applies to (**i**–**l**); the scale bar of (**m**) applies to (**n**–**r**).

*Parasitic* on leaves and stems of Poaceae. Tar spots 2–4 mm wide, on both sides of the leaf, fusiform, oblong, black, shiny. **Sexual morph**: *Ascomata* 50–290 μm high, 180–570 μm diam ($\bar{x}$ = 195 × 260 μm, n = 15), subglobose, ellipsoidal, fusiform or irregular, like black nevus, domed above the leaf surface, scattered, sometimes gregarious, black and slightly shiny, unicellular or multilocular. Ostiole 50–55 μm high, 40–48 μm diam ($\bar{x}$ = 52 × 44 μm, n = 15), circular, brown, located in the center of the ascomata. *Peridium* 26–53 μm wide ($\bar{x}$ = 36 μm, n = 25), an outer region composed of brown to dark brown cells of *textura angularis*, the internal structure comprises multiple layers of hyaline, thin walls, flattened fungal cells. *Paraphyses* 2–3 μm wide ($\bar{x}$ = 2.5 μm, n = 25), hyaline, filiform, unbranched, septate, not constricted at septate, surpassing the asci in length, with apices that taper, emanating from the inner basal and lateral walls. *Asci* 83–105 × 7–9 μm ($\bar{x}$ = 93 × 8 μm, n = 35), 8-spored, cylindrical, straight or curved, short to medium pedicellate, apex obtuse to rounded, hyaline. *Ascospores* 10–13 × 5–7 μm ($\bar{x}$ = 12 × 6 μm, n = 40), uniseriate, sometimes overlapping and oblique, ovoid to ellipsoidal, aseptate, smooth, hyaline, with one large guttule. **Asexual morph**: Undetermined.

*Host distribution*: Various Poaceae plant genera, such as *Agropyron*, *Brachypodium*, *Chloris*, *Eulalia*, *Festuca* and *Imperata* [14,40].

*Material examined*: China, Sichuan Province, Aba Tibetan Qiang Autonomous Prefecture, Li County, Miyaluo, 31°43′7″ N, 102°48′19″ E, elevation 3500 m, on stems and leaves of *Imperata cylindrica* (Poaceae), 19 October 2021, P.W. Su, MYL295 (HUEST 24.0131); ibid., on stems and leaves of *Festuca rubra* (Poaceae), MYL293. Ganzi Tibetan Autonomous Prefecture, Luding County, Hualin Village, 29°43′44″ N, 102°17′38″ E, elevation 2065 m, on stems and leaves of *Imperata cylindrica* (Poaceae), 17 November 2022, P.W. Su, HLC73. Guangyuan City, Jiange County, Lanmaqiang, 32°0′42″ N, 105°24′37″ E, elevation 655 m, on stems and leaves of *Calamagrostis epigeios* (Poaceae), 24 May 2023, P.W. Su, LMQ1.

*Notes*: *Phyllachora graminis* was introduced by Fuckel in 1870 and recognized as the type of the genus, which is widely parasitic on various grasses [40]. The phylogenetic tree indicates that our four specimens (HUEST 24.0131, MYL293, HLC73, and LMQ1) are grouped with other *P. graminis* specimens (Figure 1). Morphological comparisons show no significant differences between our newly collected specimens and other previously reported *P. graminis* specimens (Table 2). Based on thorough analysis, HUEST 24.0131, MYL293, HLC73, and LMQ1 can be classified within *P. graminis*.

#### 3.2.5. *Phyllachora luzhouensis* P.W. Su & Maharachch., sp. Nov. Figure 6

*MycoBank*: MB 852910

*Etymology*: Name reflects Luzhou, the city where the fungus was collected.

*Parasitic* on leaves and stems of *Eleusine indica* and *Chloris virgata*. Tar spots 1–2 mm wide, subcircular, rounded to oblong, black, carbonaceous. **Sexual morph**: *Ascomata* 140–360 μm high, 145–560 μm diam ($\bar{x}$ = 230 × 345 μm, n = 15), permeating the leaf tissue, encompassing its total thickness, domed above the leaf surface, subglobose, ellipsoidal to irregular, ostiole inconspicuous, scattered, solitary to gregarious, black, irregularly multilocular. *Peridium* 18–63 μm wide ($\bar{x}$ = 36 μm, n = 15), composed of brown to dark brown cells of *textura angularis* with slender, darker cells of outer layer, and large, slightly paler cells of interlayer. *Paraphyses* 1.5–3.5 μm wide ($\bar{x}$ = 2.5 μm, n = 20), hyaline, plentiful, filamentous, and lacking branches, extending beyond the asci with apices that narrow, originating from the inner basal and lateral walls. *Asci* 71–132 × 7–10 μm ($\bar{x}$ = 95 × 8 μm, n = 35), 8-spored, long and cylindrical, short to medium pedicellate, apex obtuse to rounded, hyaline. *Ascospores* 9–14 × 4–7 μm ($\bar{x}$ = 12 × 6 μm, n = 40), uniseriate, sometimes overlapping and oblique, ovoid, fusiform to ellipsoidal, acute at the ends, aseptate, hyaline, with one large guttule. **Asexual morph**: Undetermined.

**Figure 6:**
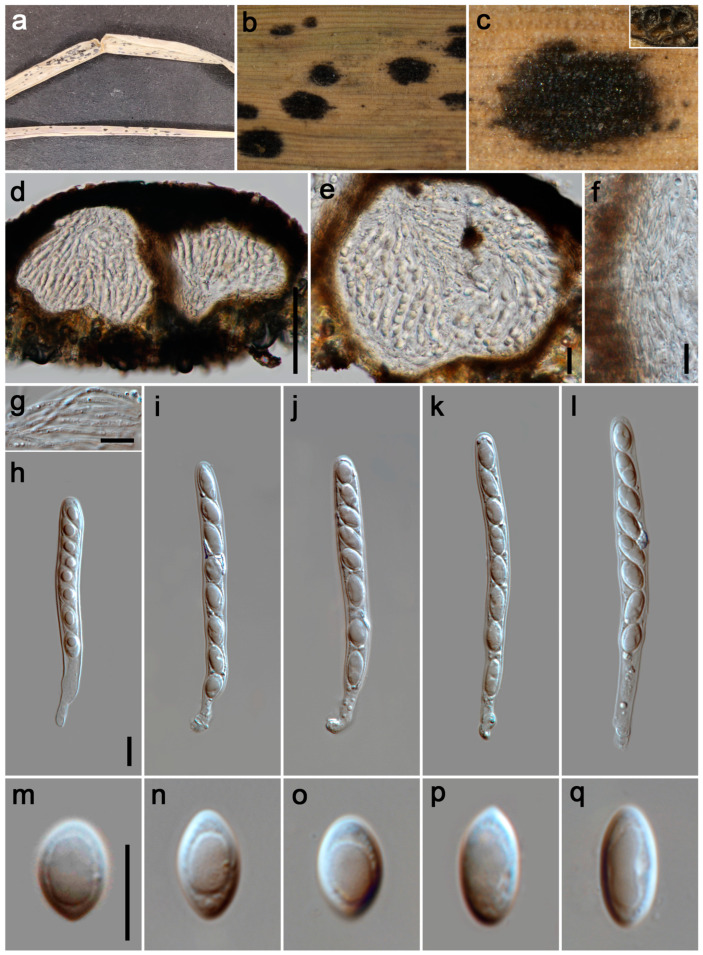
***Phyllachora luzhouensis*** (HKAS 135173, holotype). (**a**) Black spots on *Eleusine indica* (Poaceae); (**b**,**c**) Stromata and horizontal section of ascomata; (**d**,**e**) Vertical section of ascomata; (**f**) Peridium; (**g**) Paraphyses; (**h**–**l**) Asci; (**m**–**q**) Ascospores. Scale bars: 100 μm (**d**), 20 μm (**e**), 10 μm (**f**–**h**,**m**). Scale bar of (**h**) applies to (**i**–**l**); Scale bar of (**m**) applies to (**n**–**q**).

*Host distribution*: *Chloris virgata*, *Eleusine indica*.

*Material examined*: China, Sichuan Province, Luzhou City, Lu County, Xingguang Village, 29°5′55″ N, 105°27′38″ E, elevation 295 m, on stems and leaves of *Eleusine indica* (Poaceae), 30 March 2023, P.W. Su, XGC5 (HKAS 135173, holotype; HUEST 24.0133, isotype); ibid., on stems and leaves of *Chloris virgata* (Poaceae), XGC6.

*Notes:* In the multi-gene phylogenetic tree (Figure 1), our specimens HUEST 24.0133 and XGC6 form a distinct clade with high statistical support values (100% ML/1.00 PP), closely related to *Phyllachora jiaensis* (IFRD9448), *P. virgatae* (IFRD9447), *P. chloridis* (MFLU 15-0173), and *P. siamensis* (CMU TAR08). Detailed morphological comparisons reveal that HUEST 24.0133 differs from these species in the size of the asci as well as the shape and size of the ascospores (Table 2). Given the significant differences in morphology and molecular data, we propose the specimens HUEST 24.0133 and XGC6 as a new species, *Phyllachora luzhouensis*.

#### 3.2.6. *Phyllachora palmifoliae* P.W. Su & Maharachch., sp. Nov. Figure 7

*MycoBank*: MB 852911

*Etymology*: Name reflects the epithet of the host plant, *Setaria palmifolia*, from where the fungus was collected.

*Parasitic* on leaves and stems of *Setaria palmifolia*. Tar spots 2–4 mm wide, subcircular, oblong or irregular, black, shiny, carbonaceous, sometimes yellow to brown stripe at the edge of tar spots. **Sexual morph**: *Ascomata* 155–345 μm high, 170–585 μm diam ($\bar{x}$ = 250 × 375 μm, n = 15), distributed throughout the leaf tissue, spanning its full thickness, like black nevus, domed above the leaf surface, subglobose or ellipsoidal, ostiole inconspicuous, scattered, solitary to gregarious, black, unicellular or multilocular. *Peridium* 31–58 μm wide ($\bar{x}$ = 45 μm, n = 15), an outer region composed of brown to black cells of *textura angularis*, internal layers made up of various hyaline, thin-walled strata, flattened fungal cells. *Paraphyses* 1.5–3 μm wide ($\bar{x}$ = 2 μm, n = 20), hyaline, a multitude of slender, non-branching filaments, exceeding the length of the asci, featuring tapering apices that emerge from the inner basal and lateral walls. *Asci* 110–175 × 9–19 μm ($\bar{x}$ = 142 × 13 μm, n = 35), 8-spored, elongated and cylindrical, either straight or curved, with a pedicel length ranging from short to medium, and an apex that is either obtuse or rounded, exhibiting hyaline. *Ascospores* 12–17 × 5–8 μm ($\bar{x}$ = 15 × 7 μm, n = 40), uniseriate, oblique, sometimes irregularly arranged, ovoid, fusiform to ellipsoidal, aseptate, hyaline, with a gelatinous sheath, one large guttule. **Asexual morph**: Undetermined.

*Host distribution*: *Setaria palmifolia*.

*Material examined*: China, Sichuan Province, Meishan City, Danling County, Longhu Mountain, 30°3′52″ N, 103°28′45″ E, elevation 623 m, on the leaves of *Setaria palmifolia* (Poaceae), 11 July 2022, P.W. Su, LHS13 (HKAS 135170, holotype; HUEST 24.0128, isotype); ibid., LHS6, LHS21.

*Notes:* The phylogenetic tree (Figure 1) shows that the specimens HKAS 135170, LHS6, and LHS21 cluster together, forming a distinct evolutionary branch with strong statistical support (96% ML/0.97 PP). The BLASTn analysis reveals that the ITS sequence of HKAS 135170 most closely matches *Phyllachora* sp. MHYAU 120, with only 96% identity (489/509, six gaps). MHYAU 120 has not yet been published, with only the sequence data available in the GenBank database. Furthermore, morphological comparisons with closely related species indicate significant differences in the size of the asci and ascospores between HUEST 24.0128 and these species. Therefore, considering the differences in morphology and phylogeny, we describe the newly collected specimens HKAS 135170, LHS6, and LHS21 as a new species, *Phyllachora palmifoliae*.

**Figure 7:**
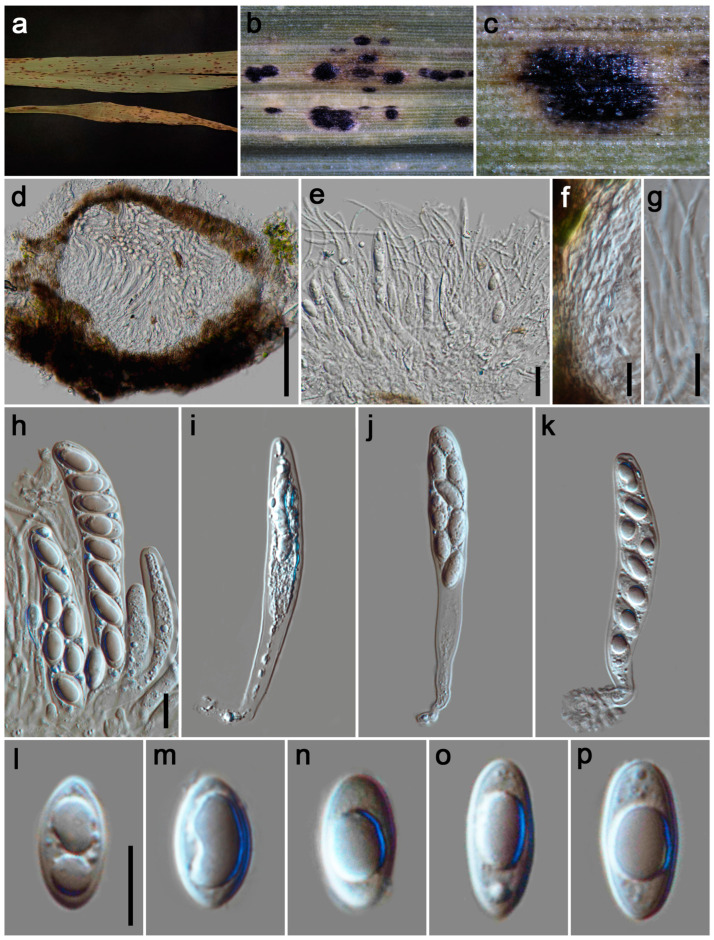
***Phyllachora palmifoliae*** (HKAS 135173). (**a**) Black spots on *Setaria palmifolia* (Poaceae); (**b**,**c**) Stromata; (**d**) Vertical section of ascomata; (**e**) Asci and paraphyses; (**f**) Peridium; (**g**) Paraphyses; (**h**–**k**) Asci; (**l**–**p**) Ascospores. Scale bars: 100 μm (**d**), 20 μm (**e**), 10 μm (**f**–**h**,**l**). Scale bar of (**h**) applies to (**i–k**); Scale bar of (**l**) applies to (**m**–**p**).

#### 3.2.7. *Phyllachora panicicola* Dayar. & K.D. Hyde, Mycosphere 8(10): 1610 (2017). Figure 8

*MycoBank*: MB 552806

**Figure 8:**
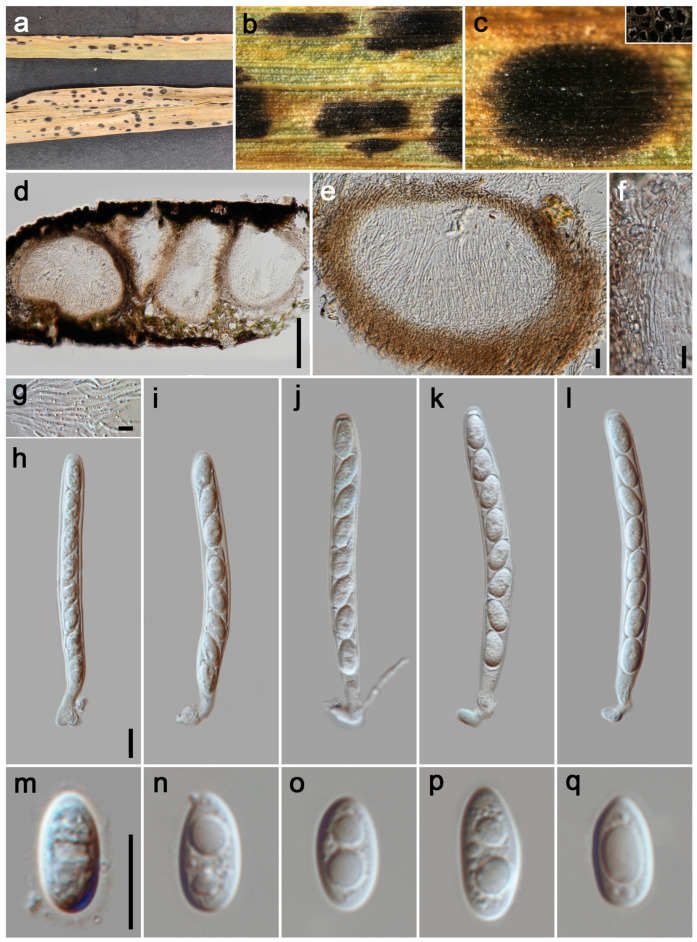
***Phyllachora panicicola*** (HUEST 24.0129). (**a**) Black spots on *Calamagrostis epigeios* (Poaceae); (**b**,**c**) Stromata and horizontal section of ascomata; (**d**,**e**) Vertical section of ascomata; (**f**) Peridium; (**g**) Paraphyses; (**h**–**l**) Asci; (**m**–**q**) Ascospores. Scale bars: 100 μm (**d**), 20 μm (**e**), 10 μm (**f**–**h**,**m**). Scale bar of (**h**) applies to (**i**–**l**); Scale bar of (**m**) applies to (**n**–**q**).

*Parasitic* on leaves and stems of Poaceae. Tar spots 1–3 mm wide, subcircular, rounded to oblong, black, carbonaceous, sometimes pale yellow to yellow stripe at the edge of tar spots. **Sexual morph**: *Ascomata* 175–330 μm high, 235–600 μm diam ($\bar{x}$ = 235 × 380 μm, n = 20), infused throughout the leaf tissue, covering its entire thickness, like black nevus, domed above the leaf surface, subglobose or ellipsoidal, ostiole inconspicuous, scattered, solitary to gregarious, black, multilocular. *Peridium* 29–67 μm wide ($\bar{x}$ = 40 μm, n = 15), an outer region composed of brown to black cells of *textura angularis*, internal configurations consist of numerous hyaline, thinly walled layers, flattened fungal cells. *Paraphyses* 1.5–3 μm wide ($\bar{x}$ = 2 μm, n = 20), hyaline, a multitude of slender, non-branching filaments, exhibiting a length greater than that of the asci, with apices that gradually narrow, stemming from the basal and lateral inner walls. *Asci* 86–145 × 8–11 μm ($\bar{x}$ = 105 × 9 μm, n = 35), 8-spored, cylindrical, short to medium pedicellate, apex obtuse to rounded, hyaline. *Ascospores* 10–14 × 5–7 μm ($\bar{x}$ = 12 × 6 μm, n = 40), uniseriate, sometimes overlapping and oblique, ovoid to ellipsoidal, aseptate, hyaline, with guttules and a gelatinous sheath. **Asexual morph**: Undetermined.

*Host distribution*: *Calamagrostis epigeios*, *Imperata cylindrica*, *Panicum* sp. [4].

*Material examined*: China, Sichuan Province, Luzhou City, Luxian County, Xingguang Village, 29°5′55″ N, 105°27′38″ E, elevation 295 m, on the stems and leaves of *Calamagrostis epigeios* and *Imperata cylindrica* (Poaceae), 30 March 2023, P.W. Su, XGC2 (HUEST 24.0129); ibid., XGC3, XGC4. Ganzi Tibetan Autonomous Prefecture, Luding County, Hualin Village, 29°43′44″ N, 102°17′38″ E, elevation 2065 m, on the stems and leaves of *Calamagrostis epigeios* and *Imperata cylindrica* (Poaceae), 17 November 2022, P.W. Su, HLC1; ibid., HLC9, HLC14.

*Notes*: In the phylogenetic tree (Figure 1), our six specimens (HUEST 24.0129, XGC3, XGC4, HLC1, HLC9, HLC14) are clustered with the known species *P. panicicola* (MFLU 16-2979). Morphological comparisons (Table 2) show no significant differences between our specimens and *P. panicicola*. Based on morphological diagnoses and phylogenetic analyses, our six specimens are identified as *P. panicicola*, representing the first records of this species from Calamagrostis epigeios and *Imperata cylindrica*.

#### 3.2.8. *Phyllachora xinpingensis* H.X. Wu & J.C. Li, in Li, Wu & Song, Phytotaxa 578(3): 279 (2023)

= *Phyllachora yuanjiangensis* H.X. Wu & J.C. Li, in Li, Wu & Song, Phytotaxa 578(3): 282 (2023)

*Notes*: *Phyllachora yuanjiangensis* and *P. xinpingensis* were introduced in the same study by Li et al. [41]. Here, we synonymized *P. yuanjiangensis* under *P. xinpingensis* based on morphological and molecular evidence, as well as the lack of host specificity within the genus. Morphologically, the differences highlighted in the original description, such as ascus length, are minor and fall within the natural variation observed in *P. xinpingensis*. The molecular phylogenetic analyses in the current study also demonstrate that the two taxa cluster as a single species (Figure 1), providing strong evidence of their conspecificity. Furthermore, the justification for *P. yuanjiangensis* as a distinct species relied on its association with a specific host plant, yet previous research [42] and this study confirm that species within *Phyllachora* are not strictly host-specific. These findings collectively support the conclusion that *P. yuanjiangensis* does not represent a separate taxon but is conspecific with *P. xinpingensis*.

#### 3.2.9. *Phyllachora wenchuanensis* P.W. Su & Maharachch., sp. Nov. Figure 9

*MycoBank*: MB 852912

*Etymology*: Name reflects Wenchuan, the place where the fungus was collected.

*Parasitic* on leaves and stems of Poaceae. Tar spots 1–2 mm wide, fusiform, oblong, black, shiny. **Sexual morph:** *Ascomata* 160–225 μm high, 200–255 μm diam ($\bar{x}$ = 175 × 225 μm, n = 20), infused throughout the leaf tissue, covering its entire thickness, like black nevus, domed above the leaf surface, subglobose or ellipsoidal, ostiole inconspicuous, scattered, solitary to gregarious, black, unicellular or multilocular. *Peridium* 26–63 μm wide ($\bar{x}$ = 43 μm, n = 15), an outer region composed of brown cells of *textura angularis*, internal configurations consist of numerous hyaline, thinly walled layers, flattened fungal cells. *Paraphyses* 1.5–2.5 μm wide ($\bar{x}$ = 2 μm, n = 20), hyaline, a multitude of slender, non-branching filaments, exhibiting a length greater than that of the asci, with apices that gradually narrow, stemming from the basal and lateral inner walls. *Asci* 75–115 × 7–10 μm ($\bar{x}$ = 105 × 9 μm, n = 35), 8-spored, cylindrical, short to medium pedicellate, apex obtuse to rounded, hyaline. *Ascospores* 10–14 × 5–7 μm ($\bar{x}$ = 12 × 6 μm, n = 40), uniseriate, oblique, ovoid or ellipsoidal, aseptate, hyaline, one guttule. **Asexual morph:** Undetermined.

*Host distribution*: *Calamagrostis epigeios*, *Elymus dahuricus*, *Imperata cylindrica*, *Lolium perenne*, *Panicum virgatum*.

*Material examined*: China, Sichuan Province, Aba Tibetan and Qiang Autonomous Prefecture, Wenchuan County, Damengou, 31°34′2″ N, 103°31′21″ E, elevation 1873 m, on the stems and leaves of *Panicum virgatum* (Poaceae), 7 July 2023, P.W. Su, DMG1 (HKAS 135174, holotype; HUEST 24.0134, isotype); ibid., DMG2. Ganzi Tibetan Autonomous Prefecture, Luding County, Hualin Village, 29°43′44″ N, 102°17′38″ E, elevation 2065 m, on the stems and leaves of *Calamagrostis epigeios* and *Imperata cylindrica* (Poaceae), 17 November 2022, P.W. Su, HLC4; ibid., HLC5, HLC20, HLC50. Aba Tibetan and Qiang Autonomous Prefecture, Wenchuan County, Xiqiang Valley, 31°29′27″ N, 103°37′1″ E, elevation 1500 m, on the stems and leaves of *Lolium perenne* (Poaceae), 20 October 2021, P.W. Su, XQDXG258; ibid., XQDXG286. Aba Tibetan and Qiang Autonomous Prefecture, Li County, Puxi Village, 31°29′19″ N, 103°16′52″ E, elevation 2412 m, on the stems and leaves of various Poaceae species (*Calamagrostis epigeios, Elymus dahuricus, Imperata cylindrica, Lolium perenne, Panicum virgatum*), P.W. Su, PXC1–PXC24.

*Note:* The phylogenetic tree (Figure 1) indicates that our 32 specimens cluster together and are closely related to *P. panicicola* (MFLU 16-2979), *P. sandiensis* (IFRD9446), and *P. flaccidudis* (IFRD9445). Our collected specimens exhibit similar morphological characteristics in the shape of asci, as seen in *P. panicicola*, *P. sandiensis*, and *P. flaccidudis*. However, *P. wenchuanensis* differs from *P. panicicola* in having shorter asci (74.81–115.06 × 7.76–10.52 vs. 110–130 × 10–14) [4]. Additionally, the BLASTn analysis (Table 3) of the ITS sequence of HKAS 135174 reveals 97% identity (512/527, seven gaps) with *P. sandiensis* (IFRD9446) and 96% identity (506/526, 7 gaps) with *P. flaccidudis* (IFRD9445). Therefore, given the differences in morphology and phylogeny, we describe the newly collected specimens as a new species.

**Figure 9:**
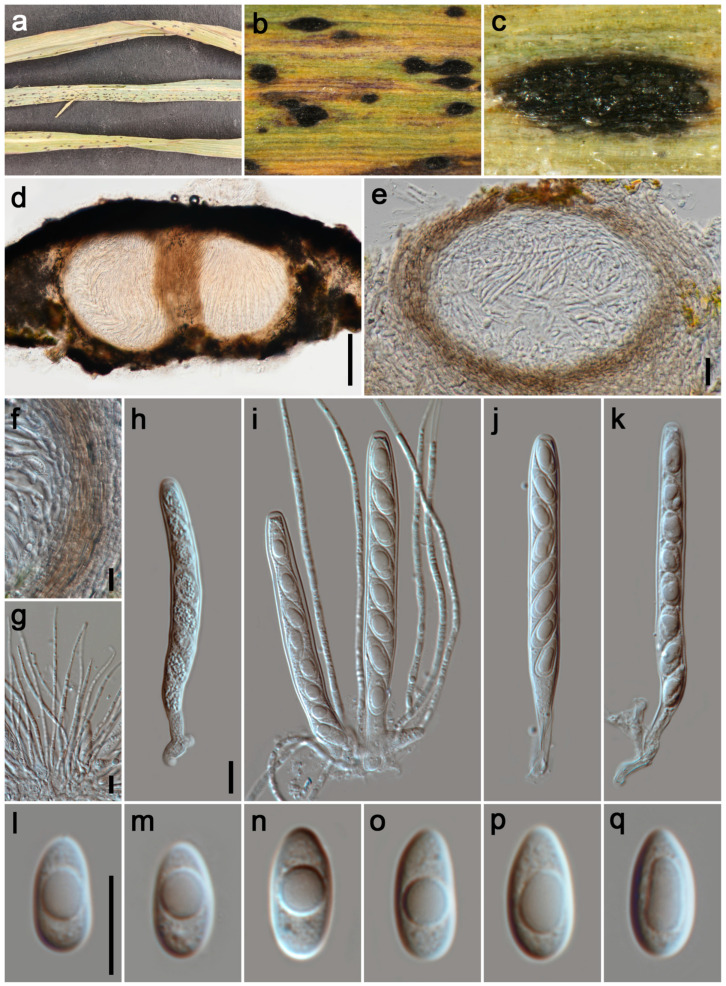
***Phyllachora wenchuanensis*** (HKAS 135174, holotype). (**a**) Black spots on *Panicum virgatum* (Poaceae); (**b**,**c**) Stromata; (**d**,**e**) Vertical section of ascomata; (**f**) Peridium; (**g**) Paraphyses; (**h**–**k**) Asci; (**l**–**q**) Ascospores. Scale bars: 100 μm (**d**), 20 μm (**e**), 10 μm (**f**–**h**,**l**). Scale bar of (**h**) applies to (**i**–**k**); Scale bar of (**l**) applies to (**m**–**q**).

**Table 2:** Morphological comparison of new species and related species of *Phyllachora*.

Species	Voucher	Hosts	Asci (µm)	Ascospores (µm)		Reference
				Size	Shape	
*P. arthraxonis*	HMAS 4306	*Arthraxon hispidus* (Arthraxon)	58–83 × 6–16	6–14 × 5–7	ellipsoidal	[14]
*P. arthraxonis* ^T^	-	*Arthraxon ciliaris* (Arthraxon)	35–45 × 8–12	8–11 × 4–5	ellipsoidal, ovoid	[38]
*P. chloridis* ^T^	MFLU 15-0173	*Chloris* sp. (Poaceae)	50–72 × 6–8	8–12 × 3.5–4.8	fusiform to oval	[4]
*P. chongzhouensis* ^T^	SICAU 24-0044	*Phragmites australis* (Poaceae)	86–142 × 14–29	13–28 × 9–15	ellipsoidal, ovoid	[16]
*P. chongzhouensis*	HUEST 24.0137	*Pennisetum purpureum* (Poaceae)	92–163 × 17–31	17–25 × 10–15	subglobose to ellipsoidal	This study
*P. chrysopogonicola* ^T^	MFLU 16-2096	*Chrysopogon zizanioides* (Poaceae)	70–122 × 12.5–15	20–26 (–30) × 6–8	unicellular to papillate to turbinate, clavate,	[43]
*P. cylindrica* ^T^	HKAS 135171	*Imperata cylindrica* (Poaceae)	80–128 × 10–16	11–15 × 6–8	tear-shaped, ovoid to ellipsoidal	This study
*P. festucae* ^T^	HKAS 135172	*Festuca elata* (Poaceae)	135–222 × 12–18	17–27 × 8–12	long ellipsoidal to fusiform	This study
*P. graminis*	HUEST 24.0131	*Imperata cylindrica* (Poaceae)	83–105 × 7–9	10–13 × 5–7	ovoid to ellipsoidal	This study
*P. graminis*	SICAU 24-005	*Lolium perenne* (Poaceae)	54–101 × 6–10	8–15 × 4–7	ellipsoidal	[16]
*P. graminis* ^T^	-	*Elymus* sp. (Poaceae)	78–80 × 7–8	8–12 × 4–5	oval to ovoid or ovoid	[14,40]
*P. keralensis*	MHYAU 13716	*Apluda mutica* (Poaceae)	48.7–79.1 × 10.7–13.6	9.2–12.8 × 5.6–6.9	ellipsoid or oblong	[44]
*P. keralensis* ^T^	-	*Apluda mutica* (Poaceae)	52–68 × 7–9.5	10–14 × 5–6.5	ellipsoid	[39]
*P. luzhouensis* ^T^	HKAS 135173	*Eleusine indica* (Poaceae)	71–132 × 7–10	9–14 × 4–7	ovoid, fusiform to ellipsoidal	This study
*P. palmifoliae* ^T^	HKAS 135170	*Setaria palmifolia* (Poaceae)	110–175 × 9–19	12–17 × 5–8	ovoid, fusiform to ellipsoidal	This study
*P. panicicola*	HUEST 24.0129	*Calamagrostis epigeios* (Poaceae)	86–145 × 7–11	10–14 × 5–7	ovoid to ellipsoidal	This study
*P. panicicola* ^T^	MFLU 16-2979	*Panicum* sp. (Poaceae)	110–130 × 10–14	14–16 × 6–8	ellipsoidal, rounded at the ends	[4]
*P. flaccidudis* ^T^	IFRD9445	*Cenchrus flaccidus* (Poaceae)	80–110 × 7–10	11–13 × 4–7	drop shape, oval to ellipse, rounded at the ends	[13]
*P. sandiensis* ^T^	IFRD9446	*Cenchrus flaccidus* (Poaceae)	92–126 × 7–10	10–14 × 6–7	drop shape, oval to ellipse	[13]
*P. siamensis* ^T^	CMU TAR08	*Eleusine indica* (Poaceae)	62–105 × 7–10	10–13 × 5–6	unicellular to ovate	[42]
*P. wenchuanensis* ^T^	HKAS 135174	*Panicum virgatum* (Poaceae)	75–115 × 7–10	10–14 × 5–7	ovoid or ellipsoidal	This study

^T^ Type species. “-” not available.

**Table 3:** Comparison of ITS sequence similarity among some species of *Phyllachora*.

ITS (%)	*P. panicicola* MFLU 16-2979	*P. sandiensis* IFRD9446	*P. flaccidudis* IFRD9445	*P. panicicola* HUEST 24.0129	*P. wenchuanensis* HKAS 135174
*P. panicicola* MFLU 16-2979	100				
*P. sandiensis* IFRD9446	97.9	100			
*P. flaccidudis* IFRD9445	96.89	99.03	100		
*P. panicicola* HUEST 24.0129	99.81	97.7	96.57	100	
*P. wenchuanensis* HKAS 135174	97.7	97.15	96.20	97.89	100

## 4. Discussion

Between 2019 and 2023, we conducted surveys on the fungal diversity of *Phyllachora* in Sichuan Province, collecting 70 specimens and identifying eight species, including five new ones. These findings underscore the significant diversity of *Phyllachora* in the region. However, due to the province’s vast geographical area, this study primarily concentrated on central Sichuan, leaving several crucial ecological zones unexplored, such as the mountainous regions of western Sichuan, the temperate forests in the northeast, and the subtropical areas in the south. To gain a more comprehensive understanding of the diversity of *Phyllachora* in Sichuan and to potentially uncover additional undiscovered species, future research should prioritize expanding the sampling range, with a particular focus on conducting thorough investigations in the southern regions, including the valleys of the Dadu River and the hills of Yunnan–Guizhou Plateau.

According to *Flora Fungorum Sinicorum*, *Vol. 46*: *Phyllachora*, as of 2009, 96 species of *Phyllachora* have been reported in China, including 26 new species, primarily found in the southern regions, namely Yunnan, Guangxi, Guangdong, and Sichuan provinces [14]. Since then, only 17 new species of *Phyllachora* have been identified in China, with molecular data available for 15 species [4,13,15,16,20,44,45,46,47]. Among these, Yunnan Province has been the most extensively studied, with the discovery of seven new species: *P. dendrocalami-hamiltoniicola*, *P. dendrocalami-membranacei*, *P. isachnicola*, *P. panicicola*, *P. sphaerocaryi*, *P. xinpingensis*, and *P. yuanjiangensis*. Additionally, four new species (*P. chongzhouensis*, *P. heteroclada*, *P. huiliensis*, *P. neidongensis*) were found in Sichuan Province, two in Hainan Province (*P. hainanensis* and *P. jianfengensis*), two in Shaanxi Province (*P. jiaensis* and *P. sandiensis*), and two in Shanxi Province (*P. flaccidudis* and *P. virgatae*).

Despite a significant number of species reported, the taxonomy of *Phyllachora* remains fraught with several unresolved issues that require urgent attention. One of the most pressing challenges is the long-standing assumption of host specificity, which has traditionally been used to define species within this genus [42]. Although *Phyllachora* species have often been regarded as host-specific, present results indicate this assumption is largely flawed. Furthermore, previous results showed that, despite being phylogenetically distinct, *P. siamensis* and *P. chloridis* were isolated from the same host genus (*Chloris* sp.) [43,48]. This lack of host specificity questions the accuracy of previous species delineations based solely on host–plant relationships. Thus, the traditional method of classifying species by their host associations has likely exaggerated the species count within the genus. For example, many *Phyllachora* species recorded in databases such as Index Fungorum and MycoBank rely on historical descriptions associated with host specificity rather than comprehensive taxonomic analysis evaluations. Reassessing species boundaries requires an approach that incorporates morphological comparisons and multi-locus phylogenetic analyses epitypification. Epitypification is particularly important for older species that were described solely based on morphology without accompanying sequence data [49]. By designating epitypes with molecular data, researchers can provide a stable taxonomic framework for these species. For instance, revisiting the type species, *Phyllachora graminis*, with modern sequencing techniques could clarify its phylogenetic position and aid in resolving species complexes within the genus. Similarly, *Phyllachora maydis*, a significant pathogen responsible for tar spot disease in maize, could benefit from epitypification to standardize its identity and facilitate comparisons with closely related species.

In addition to the issue of host specificity, the genus *Phyllachora* encounters notable taxonomic challenges due to its morphological similarities with other genera, such as *Polystigma*. These two genera are often indistinguishable based solely on their morphology, with stromatal pigmentation being one of the few distinguishing features [50]. However, these subtle morphological differences complicate the reliable differentiation of species without molecular analysis. The limited availability of type sequences for *Phyllachora* in GenBank exacerbates this issue, hindering the establishment of accurate species boundaries. The scarcity of type material, coupled with the morphological plasticity of the genus, highlights the necessity for a more comprehensive revision of its taxonomy.

*Phyllachora* is an ancient and species-rich genus comprising over 900 morphologically recognized species. However, molecular data are available for only 44 species in GenBank, including the five new species identified in this study. This limitation primarily arises because the taxa are biotrophic and unable to grow in culture, making obtaining sequence data from freshly collected specimens challenging. Consequently, the taxonomy of the genus remains largely understudied. Only a limited number of studies have explored the phylogeny of *Phyllachora*, predominantly based on ITS, LSU, and SSU sequence data, which has revealed that *Phyllachora* is polyphyletic [4,11]. In this study, we observed that while ITS provided some degree of species resolution, LSU and SSU sequences showed limited power in distinguishing *Phyllachora* species (Figure S1), with both newly described and previously reported taxa clustering together. Furthermore, the limited resolution of LSU and SSU markers in our study limits the application of the concept of Genealogical Concordance Phylogenetic Species Recognition, as these regions do not provide a sufficient phylogenetic signal to resolve species-level relationships. Although we attempted to include additional protein-coding markers, such as *TEF1* and *RPB2*, PCR amplification was unsuccessful due to primer mismatches and weak amplification signals. We also further attempted whole-genome sequencing, which was complicated by contamination from the plant host DNA, highlighting the need for optimized DNA extraction techniques and novel primers tailored for *Phyllachora* phylogenetics. Therefore, we emphasize the need for further studies incorporating additional gene regions, particularly those derived from protein-coding genes, to better resolve the relationships among closely related species in the genus *Phyllachora.* Phylogenomic analysis can also be utilized to identify additional genetic markers for distinguishing taxa within species of *Sordariomycetes* [51], including those in the genus *Phyllachora*. Additionally, sequences from the type specimen of *P. graminis* should be generated to understand the species boundaries of *Phyllachora sensu stricto*.

## Figures and Tables

**Table 1:** Species information and corresponding GenBank accession numbers used in this study. The newly generated sequences are indicated in bold. Type collections are marked with “^T^”. “-” means sequence unavailable.

Species	Voucher	Location	Host	Host Family	GenBank Accession Numbers		
					LSU	ITS	SSU
*Ascovaginospora stellipala* ^T^	ATCC 200781	USA	*Carex limosa*	Cyperaceae	U85088	-	U85087
*Camarotella costaricensis*	MM-21	Panama	*Acrocomia aculeata*	Arecaceae	KX430490	KX451900	KX451851
Ca. *costaricensis*	MM-149	Panama	*Acrocomia aculeata*	Arecaceae	KX430484	KX451913	KX451863
*Coccodiella calatheae* ^T^	MP5133	Panama	*Calathea crotalifera*	Marantaceae	MF460370	MF460366	MF460376
*Co. melastomatum*	CMU78543	Venezuela	*Miconia* sp.	Melastomataceae	-	-	U78543
*Co. miconiae*	ppMP1342	Panama	*Miconia* sp.	Melastomataceae	KX430506	MF460365	KX451871
*Co. miconiicola*	SO-15	Ecuador	*Graffenrieda* sp.	Melastomataceae	MF460374	MF460369	MF460380
*Co. miconiicola*	CBMAP-H290A	Panama	*Miconia* sp.	Melastomataceae	MF460373	MF460368	MF460379
*Co. toledoi*	MM-165	Ecuador	*Miconia* sp.	Melastomataceae	KX430488	KX451917	KX451865
*Neophyllachora cerradensis* ^T^	UB21908	Brazil	*Myrcia torta*	Myrtaceae	-	KC683471	-
*N. cerradensis*	UB21823	Brazil	*Myrcia torta*	Myrtaceae	-	KC683470	-
*N. myrciae*	UB21292	Brazil	*Myrcia pallens*	Myrtaceae	-	KC683463	-
*N. myrciae*	UB22192	Brazil	*Myrcia pallens*	Myrtaceae	-	KC683476	-
*N. myrciariae* ^T^	UB21781	Brazil	*Myrciaria delicatula*	Myrtaceae	-	KC683469	-
*N. religiosa* ^T^	MFLU 23-0258	Thailand	*Ficus religiosa*	Moraceae	-	OQ821010	-
*N. religiosa*	MFLU 23-0254	Thailand	*Ficus religiosa*	Moraceae	-	OQ821006	-
*N. subcircinans*	UB09748	Brazil	*Myrtaceae*	Myrtaceae	-	KC683441	-
*N. subcircinans*	UB21347	Brazil	*Myrtaceae*	Myrtaceae	-	KC683466	-
*N. subcircinans*	UB21747	Brazil	*Myrtaceae*	Myrtaceae	-	KC683467	KC902622
*N. truncatispora*	UB14083	Brazil	*Myrcia camapuanensis*	Myrtaceae	-	KC683448	KC902614
*Phyllachora arthraxonis*	MHYAU 073	China	*Arthraxon hispidus*	Poaceae	MG269804	MG269750	-
*P. arthraxonis*	MHYAU 143	China	*Arthraxon hispidus*	Poaceae	MG269805	MG269751	MH992445
*P. arundinellae*	MHYAU 108	China	*Arundinella setosa*	Poaceae	MG269815	MG269761	MH992450
*P. arundinellae*	MHYAU 160	China	*Arundinella setosa*	Poaceae	MG269816	MG269762	-
*P. chloridis* ^T^	MFLU 15-0173	Thailand	*Chloris* sp.	Poaceae	MF197499	KY594026	MF197505
*P. chloridis*	MFLU 16-2980	Thailand	*Poaceae* sp.	Poaceae	MF197500	KY594027	MF197506
*P. chloridis*	SICAU 24-0053	China	*Chloris virgata*	Poaceae	PP785310	PP785299	PP785321
*P. chloridis*	SICAU 24-0054	China	*Chloris virgata*	Poaceae	PP785311	PP785300	PP785322
*P. chloridis-virgatae*	MHYAU 20136	China	*Chloris virgata*	Poaceae	MG356685	KY498122	-
*P. chloridis-virgatae*	MHYAU 20137	China	*Chloris virgata*	Poaceae	MG356686	KY498092	-
*P. chongzhouensis* ^T^	SICAU 24-0044	China	*Phragmites australis*	Poaceae	PP785312	PP785301	PP785323
*P. chongzhouensis*	SICAU 24-0045	China	*Phragmites australis*	Poaceae	PP785313	PP785302	PP785324
***P. chongzhouensis***	**HUEST 24.0137**	**China**	***Pennisetum purpureum***	**Poaceae**	**PP464570**	**PP472693**	**PP464728**
***P. chongzhouensis***	**LJT3**	**China**	***Pennisetum purpureum***	**Poaceae**	**PP464571**	**PP472694**	**PP464729**
***P. chongzhouensis***	**LJT4**	**China**	***Pennisetum purpureum***	**Poaceae**	**PP464572**	**PP472695**	**PP464730**
***P. chongzhouensis***	**LJT5**	**China**	***Pennisetum purpureum***	**Poaceae**	**PP464573**	**PP472696**	**PP464731**
***P. chongzhouensis***	**LJT6**	**China**	***Pennisetum purpureum***	**Poaceae**	**PP464574**	**PP472697**	**PP464732**
***P. chongzhouensis***	**LJT7**	**China**	***Pennisetum purpureum***	**Poaceae**	**PP464575**	**PP472698**	**PP464733**
***P. chongzhouensis***	**LMQ2**	**China**	***Imperata cylindrica***	**Poaceae**	**PP464577**	**PP472700**	**PP464735**
***P. chongzhouensis***	**LMQ3**	**China**	***Imperata cylindrica***	**Poaceae**	**PP464578**	**PP472701**	**PP464736**
***P. chongzhouensis***	**QQS6**	**China**	***Imperata cylindrica***	**Poaceae**	**PP464613**	**PP472736**	**PP464771**
***P. chongzhouensis***	**QQS7**	**China**	***Imperata cylindrica***	**Poaceae**	**PP464614**	**PP472737**	**PP464772**
***P. chongzhouensis***	**QQS13**	**China**	***Imperata cylindrica***	**Poaceae**	**PP464608**	**PP472731**	**PP464766**
***P. chongzhouensis***	**YM1**	**China**	***Miscanthus floridulus***	**Poaceae**	**PP464624**	**PP472747**	**PP464782**
***P. chongzhouensis***	**YM2**	**China**	***Miscanthus floridulus***	**Poaceae**	**PP464625**	**PP472748**	**PP464783**
***P. chongzhouensis***	**YM3**	**China**	***Miscanthus floridulus***	**Poaceae**	**PP464626**	**PP472749**	**PP464784**
*P. chrysopogonicola* ^T^	MFUH 16-2096	Thailand	*Chrysopogon zizanioides*	Poaceae	MF372146	MF372145	-
***P. cylindricae* ^T^**	**HKAS 135171**	**China**	***Imperata cylindrica***	**Poaceae**	**PP464615**	**PP472738**	**PP464773**
***P. cylindricae***	**SBZ3**	**China**	***Imperata cylindrica***	**Poaceae**	**PP464616**	**PP472739**	**PP464774**
***P. cylindricae***	**QQS2**	**China**	***Panicum repens***	**Poaceae**	**PP464612**	**PP472735**	**PP464770**
***P. cylindricae***	**QQS14**	**China**	***Panicum repens***	**Poaceae**	**PP464609**	**PP472732**	**PP464767**
***P. cylindricae***	**QQS15**	**China**	***Panicum repens***	**Poaceae**	**PP464610**	**PP472733**	**PP464768**
***P. cylindricae***	**QQS16**	**China**	***Panicum repens***	**Poaceae**	**PP464611**	**PP472734**	**PP464769**
*P. cynodonticola* ^T^	MFLU 16-2977	Thailand	*Cynodon* sp.	Poaceae	MF197501	KY594024	MF197507
*P. cynodonticola*	MFLU 16-2978	Thailand	*Imperata* sp.	Poaceae	MF197502	KY594025	MF197508
*P. cynodontis*	MHYAU 20043	China	*Cynodon dactylon*	Poaceae	KY498081	KY471329	MH992435
*P. cynodontis*	MHYAU 20131	China	*Cynodon dactylon*	Poaceae	KY498079	KY471327	-
*P. dendrocalami-hamiltonii* ^T^	MHYAU 221	China	*Dendrocalamus hamiltonii*	Poaceae	MK614118	-	-
*P. dendrocalami-membranacei* ^T^	MHYAU 220	China	*Dendrocalamus membranaceus*	Poaceae	MK614117	MK614102	-
*P. dendrocalami-membranacei*	MHYAU 222	China	*Dendrocalamus membranaceus*	Poaceae	MK614119	MK614103	-
*P. festucae* ^T^	HKAS 135172	China	*Festuca elata*	Poaceae	PP464617	PP472740	PP464775
*P. festucae*	SMGZ2	China	*Festuca elata*	Poaceae	PP464618	PP472741	PP464776
*P. festucae*	SMGZ3	China	*Festuca elata*	Poaceae	PP464619	PP472742	PP464777
*P. flaccidudis* ^T^	IFRD9445	China	*Cenchrus flaccidus*	Poaceae	ON072101	ON075524	ON072097
***P. graminis***	**HUEST 24.0131**	**China**	***Imperata cylindrica***	**Poaceae**	**PP464623**	**PP472746**	**PP464781**
***P. graminis***	**MYL293**	**China**	***Festuca rubra***	**Poaceae**	**PP464622**	**PP472745**	**PP464780**
***P. graminis***	**HLC73**	**China**	***Imperata cylindrica***	**Poaceae**	**PP464565**	**PP472688**	**PP464723**
***P. graminis***	**LMQ1**	**China**	***Calamagrostis epigeios***	**Poaceae**	**PP464576**	**PP472699**	**PP464734**
*P. graminis*	AF257111	Unknown	*Unknown*	Poaceae	-	AF257111	-
*P. graminis*	RoKi3084	Germany	*Arrhenatherum elatius*	Poaceae	-	-	KX451872
*P. graminis*	UME31349	Sweden	*Unknown*	Poaceae	-	-	AF064051
*P. graminis*	SICAU 24-0051	China	*Lolium perenne*	Poaceae	PP785306	PP785295	PP785317
*P. graminis*	SICAU 24-0052	China	*Lolium perenne*	Poaceae	PP785307	PP785296	PP785318
*P. heterocladae* ^T^	MFLU 18-1221	China	*Phyllostachys heteroclada*	Poaceae	MK296472	MK305902	MK296468
*P. huiliensis* ^T^	SICAU 24-0048	China	*Bothriochloa ischaemum*	Poaceae	PP785308	PP785297	PP785319
*P. huiliensis*	SICAU 24-0049	China	*Bothriochloa ischaemum*	Poaceae	PP785309	PP785298	PP785320
*P. imperatae*	MHYAU 013	China	*Imperata cylindrica*	Poaceae	MG269799	MG269745	-
*P. imperatae*	MHYAU 014	China	*Imperata cylindrica*	Poaceae	MG269800	MG269746	-
*P. indosasae*	MHYAU 125	China	*Indosasa hispida*	Poaceae	MG195662	MG195637	-
*P. isachnicola* ^T^	MHYAU 179	China	*Isachne albens*	Poaceae	MH018563	MH018561	-
*P. isachnicola*	MHYAU 180	China	*Isachne albens*	Poaceae	MH018564	MH018562	-
*P. jiaensis* ^T^	IFRD9448	China	*Chloris virgata*	Poaceae	ON075440	ON075527	ON072100
*P. keralensis*	MHYAU 20082	China	*Apluda mutica*	Poaceae	MG269792	KY498106	MH992447
*P. keralensis*	MHYAU 20083	China	*Apluda mutica*	Poaceae	MG269793	KY498088	-
***P. luzhouensis* ^T^**	**HKAS 135173**	**China**	***Eleusine indica***	**Poaceae**	**PP464582**	**PP472705**	**PP464740**
***P. luzhouensis***	**XGC6**	**China**	***Chloris virgata***	**Poaceae**	**PP464583**	**PP472706**	**PP464741**
*P. maydis*	BPI 893231	USA	*Zea mays*	Poaceae	-	KU184459	-
*P. maydis*	BPI 910560	Wisconsin	*Zea mays*	Poaceae	-	MG881846	-
*P. maydis*	C18162-3	USA	*Zea mays*	Poaceae	OL314408	OL342794	-
*P. maydis*	C18164-2	USA	*Zea mays*	Poaceae	OL314409	OL342796	-
*P. miscanthi*	MHYAU 157	China	*Miscanthus sinensis*	Poaceae	MG195668	MG195643	-
*P. miscanthi*	MHYAU 167	China	*Miscanthus sinensis*	Poaceae	MG195669	MG195644	-
*P. miscanthi*	SICAU 24-0050	China	*Miscanthus floridulus*	Poaceae	PP785305	PP785294	PP785316
*P. neidongensis*	SICAU 24-0046	China	*Themeda triandra*	Poaceae	PP785314	PP785303	PP785325
*P. neidongensis*	SICAU 24-0047	China	*Themeda triandra*	Poaceae	PP785315	PP785304	PP785326
*P. oplismeni-compositi*	MHYAU 170	China	*Oplismenus compositus*	Poaceae	MG195673	MG195648	-
***P. palmifoliae* ^T^**	**HKAS 135170**	**China**	***Setaria palmifolia***	**Poaceae**	**PP464568**	**PP472691**	**PP464726**
***P. palmifoliae***	**LHS6**	**China**	***Setaria palmifolia***	**Poaceae**	**PP464567**	**PP472690**	**PP464725**
***P. palmifoliae***	**LHS21**	**China**	***Setaria palmifolia***	**Poaceae**	**PP464569**	**PP472692**	**PP464727**
*P. panicicola* ^T^	MFLU 16-2979	China	*Panicum* sp.	Poaceae	MF197503	KY594028	MF197504
***P. panicicola***	**HUEST 24.0129**	**China**	***Calamagrostis epigeios***	**Poaceae**	**PP464579**	**PP472702**	**PP464737**
***P. panicicola***	**XGC3**	**China**	***Calamagrostis epigeios***	**Poaceae**	**PP464580**	**PP472703**	**PP464738**
***P. panicicola***	**XGC4**	**China**	***Imperata cylindrica***	**Poaceae**	**PP464581**	**PP472704**	**PP464739**
***P. panicicola***	**HLC1**	**China**	***Calamagrostis epigeios***	**Poaceae**	**PP464559**	**PP472682**	**PP464717**
***P. panicicola***	**HLC9**	**China**	***Calamagrostis epigeios***	**Poaceae**	**PP464566**	**PP472689**	**PP464724**
***P. panicicola***	**HLC14**	**China**	***Imperata cylindrica***	**Poaceae**	**PP464560**	**PP472683**	**PP464718**
*P. pogonatheri*	MHYAU 070	China	*Pogonatherum crinitum*	Poaceae	MG269801	MG269747	-
*P. pogonatheri*	MHYAU 071	China	*Pogonatherum paniceum*	Poaceae	MG269802	MG269748	-
*P. qualeae*	UB21159	Brazil	*Qualea multiflora*	Vochysiaceae	-	KU682781	-
*P. qualeae*	UB21771	Brazil	*Qualea multiflora*	Vochysiaceae	-	KU682780	-
*P. sacchari-spontanei*	MHYAU 087	China	*Saccharum spontaneum*	Poaceae	MG195670	MG195645	-
*P. sandiensis* ^T^	IFRD9446	China	*Cenchrus flaccidus*	Poaceae	ON075528	ON075525	ON072098
*P. siamensis* ^T^	CMU TAR08	Thailand	*Eleusine indica*	Poaceae	MZ749659	MZ749653	MZ749661
*P. siamensis*	CMU TAR12	Thailand	*Chloris barbata*	Poaceae	MZ749660	MZ749654	MZ749662
*P. sinobambusae*	MHYAU 085	China	*Sinobambusa tootsik*	Poaceae	MG195655	MG195630	-
*P. sphaerocaryi* ^T^	MHYAU 178	China	*Sphaerocaryum malaccense*	Poaceae	-	MH018560	-
*P. sphaerocaryi*	MHYAU 217	China	*Sphaerocaryum malaccense*	Poaceae	MK614114	MK614100	-
*P. thysanolaenae* ^T^	MFLU 16-2071	Thailand	*Thysanolaena maxima*	Poaceae	-	-	MF372147
*P. virgatae* ^T^	IFRD9447	China	*Chloris virgata*	Poaceae	ON075439	ON075526	ON072099
***P. wenchuanensis* ^T^**	**HKAS 135174**	**China**	***Panicum virgatum***	**Poaceae**	**PP464557**	**PP472680**	**PP464715**
***P. wenchuanensis***	**DMG2**	**China**	***Panicum virgatum***	**Poaceae**	**PP464558**	**PP472681**	**PP464716**
***P. wenchuanensis***	**XQDXG258**	**China**	***Lolium perenne***	**Poaceae**	**PP464620**	**PP472743**	**PP464778**
***P. wenchuanensis***	**XQDXG286**	**China**	***Lolium perenne***	**Poaceae**	**PP464621**	**PP472744**	**PP464779**
***P. wenchuanensis***	**HLC4**	**China**	***Calamagrostis epigeios***	**Poaceae**	**PP464562**	**PP472685**	**PP464720**
***P. wenchuanensis***	**HLC5**	**China**	***Calamagrostis epigeios***	**Poaceae**	**PP464563**	**PP472686**	**PP464721**
***P. wenchuanensis***	**HLC20**	**China**	***Imperata cylindrica***	**Poaceae**	**PP464561**	**PP472684**	**PP464719**
***P. wenchuanensis***	**HLC50**	**China**	***Calamagrostis epigeios***	**Poaceae**	**PP464564**	**PP472687**	**PP464722**
***P. wenchuanensis***	**PXC1**	**China**	***Imperata cylindrica***	**Poaceae**	**PP464584**	**PP472707**	**PP464742**
***P. wenchuanensis***	**PXC2**	**China**	***Imperata cylindrica***	**Poaceae**	**PP464595**	**PP472718**	**PP464753**
***P. wenchuanensis***	**PXC3**	**China**	***Imperata cylindrica***	**Poaceae**	**PP464601**	**PP472724**	**PP464759**
***P. wenchuanensis***	**PXC4**	**China**	***Imperata cylindrica***	**Poaceae**	**PP464602**	**PP472725**	**PP464760**
***P. wenchuanensis***	**PXC5**	**China**	***Imperata cylindrica***	**Poaceae**	**PP464603**	**PP472726**	**PP464761**
***P. wenchuanensis***	**PXC6**	**China**	***Imperata cylindrica***	**Poaceae**	**PP464604**	**PP472727**	**PP464762**
***P. wenchuanensis***	**PXC7**	**China**	***Calamagrostis epigeios***	**Poaceae**	**PP464605**	**PP472728**	**PP464763**
***P. wenchuanensis***	**PXC8**	**China**	***Imperata cylindrica***	**Poaceae**	**PP464606**	**PP472729**	**PP464764**
***P. wenchuanensis***	**PXC9**	**China**	***Calamagrostis epigeios***	**Poaceae**	**PP464607**	**PP472730**	**PP464765**
***P. wenchuanensis***	**PXC10**	**China**	***Lolium perenne***	**Poaceae**	**PP464585**	**PP472708**	**PP464743**
***P. wenchuanensis***	**PXC11**	**China**	***Panicum virgatum***	**Poaceae**	**PP464586**	**PP472709**	**PP464744**
***P. wenchuanensis***	**PXC12**	**China**	***Elymus dahuricus***	**Poaceae**	**PP464587**	**PP472710**	**PP464745**
***P. wenchuanensis***	**PXC13**	**China**	***Calamagrostis epigeios***	**Poaceae**	**PP464588**	**PP472711**	**PP464746**
***P. wenchuanensis***	**PXC14**	**China**	***Lolium perenne***	**Poaceae**	**PP464589**	**PP472712**	**PP464747**
***P. wenchuanensis***	**PXC15**	**China**	***Lolium perenne***	**Poaceae**	**PP464590**	**PP472713**	**PP464748**
***P. wenchuanensis***	**PXC16**	**China**	***Lolium perenne***	**Poaceae**	**PP464591**	**PP472714**	**PP464749**
***P. wenchuanensis***	**PXC17**	**China**	***Elymus dahuricus***	**Poaceae**	**PP464592**	**PP472715**	**PP464750**
***P. wenchuanensis***	**PXC18**	**China**	***Lolium perenne***	**Poaceae**	**PP464593**	**PP472716**	**PP464751**
***P. wenchuanensis***	**PXC19**	**China**	***Panicum virgatum***	**Poaceae**	**PP464594**	**PP472717**	**PP464752**
***P. wenchuanensis***	**PXC20**	**China**	***Elymus dahuricus***	**Poaceae**	**PP464596**	**PP472719**	**PP464754**
***P. wenchuanensis***	**PXC21**	**China**	***Panicum virgatum***	**Poaceae**	**PP464597**	**PP472720**	**PP464755**
***P. wenchuanensis***	**PXC22**	**China**	***Panicum virgatum***	**Poaceae**	**PP464598**	**PP472721**	**PP464756**
***P. wenchuanensis***	**PXC23**	**China**	***Lolium perenne***	**Poaceae**	**PP464599**	**PP472722**	**PP464757**
***P. wenchuanensis***	**PXC24**	**China**	***Lolium perenne***	**Poaceae**	**PP464600**	**PP472723**	**PP464758**
***P. xinpingensis***	**IFRD9465**	**China**	***Chrysopogon aciculatus***	**Poaceae**	**OP359416**	**OP359398**	**-**
***P. yuanjiangensis***	**IFRD9466**	**China**	***Arundinella setosa***	**Poaceae**	**OP359417**	**OP359399**	**OP359400**
*P. yushaniae-falcatiauritae*	MHYAU 123	China	*Yushania falcatiaurita*	Poaceae	MG195656	MG195631	-
*P. yushaniae-polytrichae*	MHYAU 122	China	*Yushania polytricha*	Poaceae	MG195657	MG195632	-
*P. yushaniae-polytrichae*	MHYAU 158	China	*Yushania polytricha*	Poaceae	MG195658	MG195633	-
*Telimena bicincta*	MM-133	Costa Rica	*Picramnia antidesma*	Picramniaceae	KX430478	KX451910	KX451861

## Data Availability

All sequence data are available in NCBI GenBank following the accession numbers in the manuscript.

## Supplementary Materials

The following supporting information can be downloaded at: https://www.mdpi.com/article/10.3390/jof11030208/s1. Table S1: Detailed information of sampling sites. Figure S1: Single-gene trees. Novel isolates are indicated in red. Type isolates are in bold. The ML ultrafast bootstrap values greater than 95% are indicated at the respective nodes.
