# Supplementary figures and images for: Five New Phyllachora Species from Tar Spot Fungi on Poaceae in Sichuan China
Source: J Fungi (Basel). 2025 Mar 7;11(3):208. doi: 10.3390/jof11030208 (PMC11943046; doi:10.3390/jof11030208)

ITS

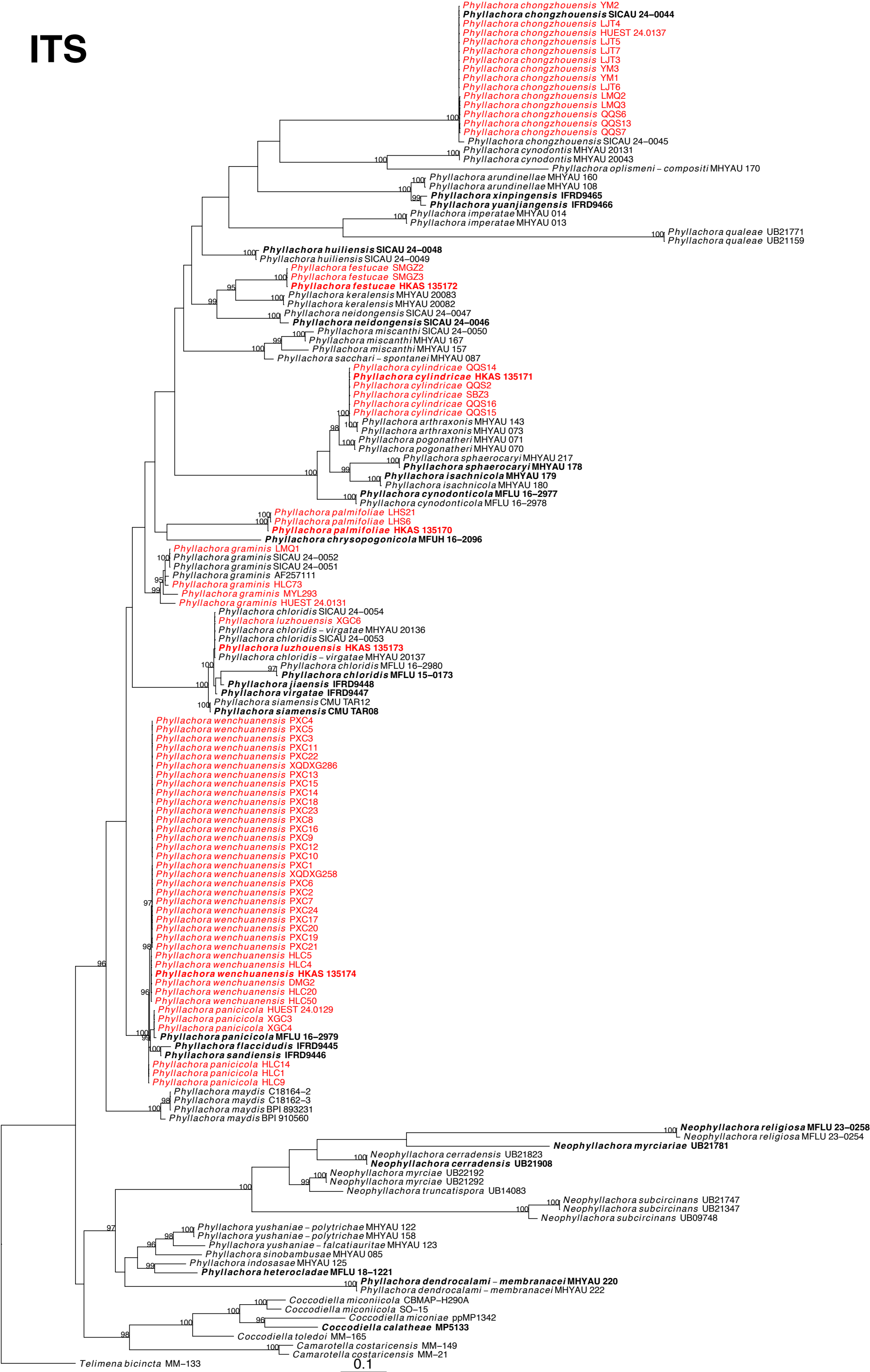

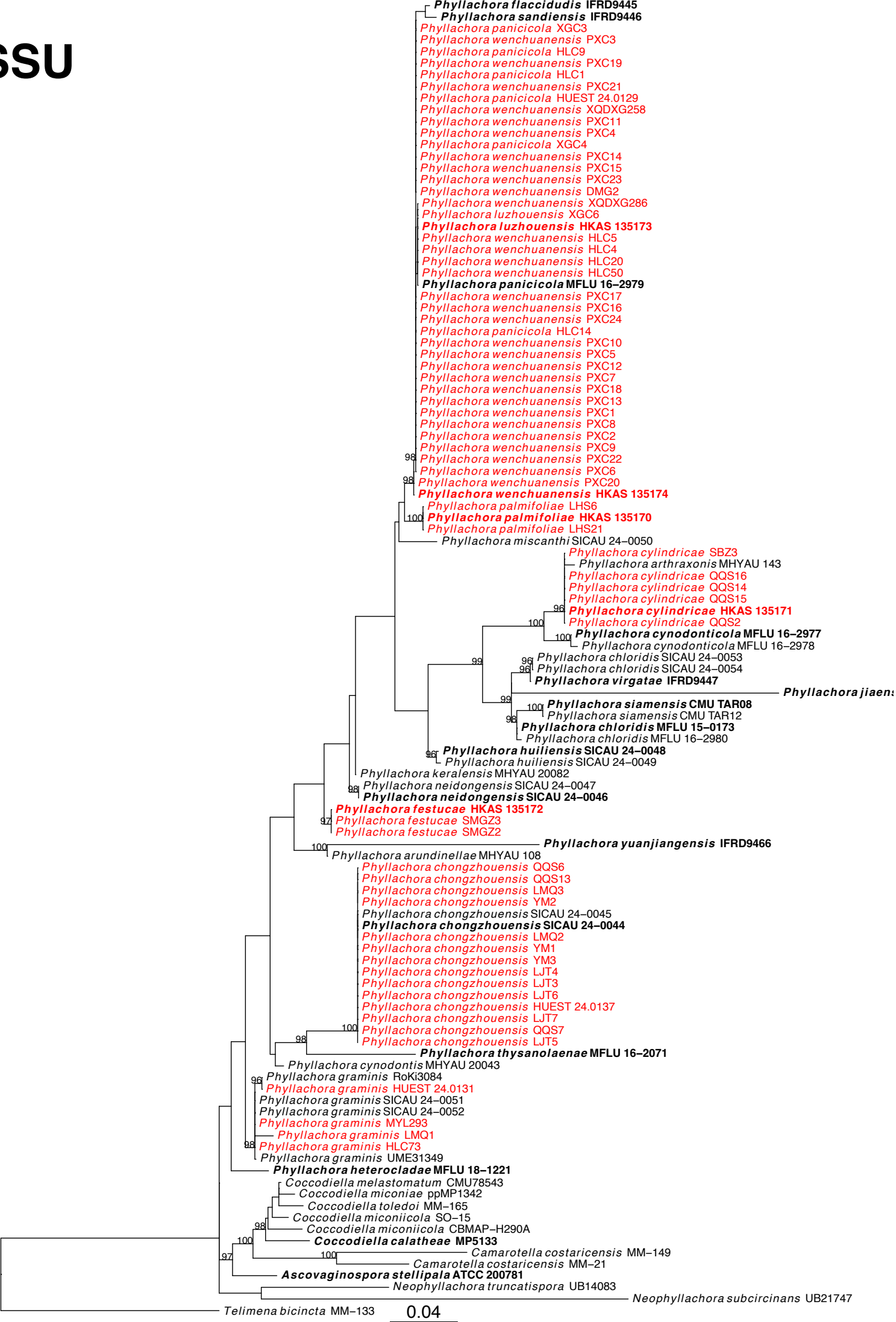

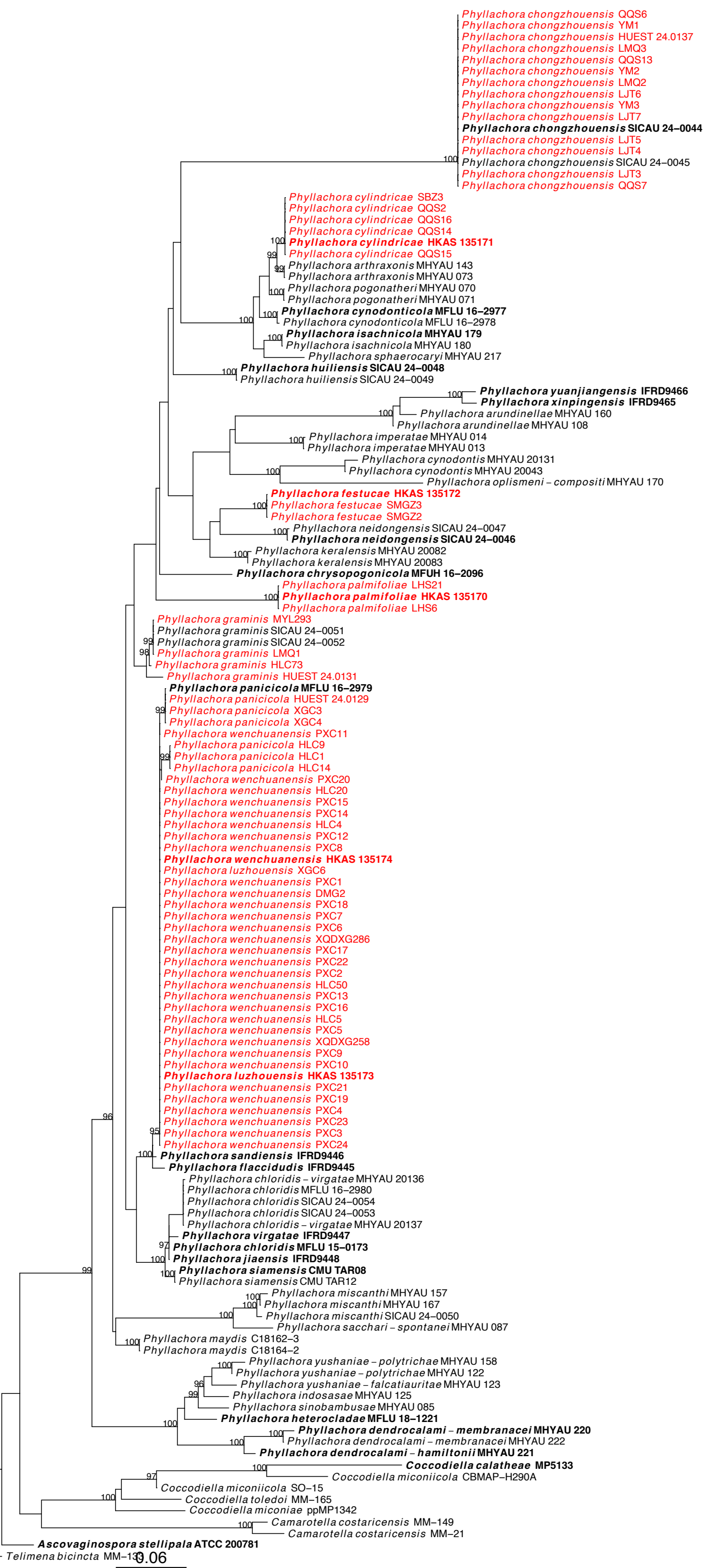

Supplement: Supplementary file 1 [file jof-11-00208-s001.zip › Figure S1.pdf]
